# Review: Genetic and protein variants of milk caseins in goats

**DOI:** 10.3389/fgene.2022.995349

**Published:** 2022-12-07

**Authors:** Siham A. Rahmatalla, Danny Arends, Gudrun A. Brockmann

**Affiliations:** ^1^ Animal Breeding and Molecular Genetics, Albrecht Daniel Thaer-Institute of Agricultural and Horticultural Sciences, Humboldt University of Berlin, Berlin, Germany; ^2^ Department of Dairy Production, Faculty of Animal Production, University of Khartoum, Khartoum North, Sudan; ^3^ Department of Applied Sciences, Northumbria University, Newcastle upon Tyne, United Kingdom

**Keywords:** milk protein, casein, goat, genetic variations, polymorphisms, nutrition, health

## Abstract

The milk casein genes in goats, are highly polymorphic genes with numerous synonymous and non-synonymous mutations. So far, 20 protein variants have been reported in goats for alpha-S1-casein, eight for beta-casein, 14 for alpha-S2-casein, and 24 for kappa-casein. This review provides a comprehensive overview on identified milk casein protein variants in goat and non-coding DNA sequence variants with some affecting the expression of the casein genes. The high frequency of some casein protein variants in different goat breeds and geographical regions might reflect specific breeding goals with respect to milk processing characteristics, properties for human nutrition and health, or adaptation to the environment. Because protein names, alongside the discovery of protein variants, go through a historical process, we linked old protein names with new ones that reveal more genetic variability. The haplotypes across the cluster of the four genetically linked casein genes are recommended as a valuable genetic tool for discrimination between breeds, managing genetic diversity within and between goat populations, and breeding strategies. The enormous variation in the casein proteins and genes is crucial for producing milk and dairy products with different properties for human health and nutrition, and for genetic improvement depending on local breeding goals.

## Introduction

Casein is the insoluble major protein component of milk comprising about 80% of the total milk protein fraction (Bhat et al., 2016). Milk caseins have a fundamental nutritional value since they provide calcium and phosphorus for bone formation and amino acid synthesis in offspring (Barth and Schlimme, 2012; Ghosh et al., 2016). Milk composition is a multifactorial complex trait. Besides genetic factors, the total protein and casein content is affected by various factors such as number of milking per day, stage of lactation, age of the animal, parity, rearing practices, seasonal components, and nutrition (Assan, 2014).

In goats, like in other mammals, four evolutionary conserved casein genes occur. The genes encoding alpha-S1-, beta-, alpha-S2-, and kappa-caseins are *CSN1S1*, *CSN2*, *CSN1S2*, and *CSN3*, respectively (Martin et al., 2002). All four genes are located in a 250 kb region between 85.978 and 86.211 Mb on *Capra hircus* chromosome 6 (Ferretti et al., 1990; Threadgill and Womack, 1990).

Caseins occur in milk in micelles which are large spherical colloidal particles. These micelles are buildup of several submicelles which are bound together by colloidal calcium phosphate (Fox and McSweeney, 1998). Alpha-S1-, α-S2-, and β-caseins, which are hydrophobic phosphate and calcium-sensitive caseins, are located inside the inner part of the micelle (Hui and Sherkat, 2005), which precipitate from raw milk upon acidification at pH 4.6 at 20°C⁰ (Park et al., 2017). Kappa-casein, the insensitive calcium protein, resides on the surface of the micelles where it stabilizes the micelles with a protruding hydrophobic chain (Guinee and O'Brien, 2010).

The most important economic effects of the milk casein proteins come from the relationship between protein quality, amount, coagulation, and techno-functional properties to produce yoghurts and cheese. Casein polymorphisms also affect the fat content, micellar size, mineralization, and sensory characteristic in milk. Therefore, casein polymorphisms play a significant role both in milk coagulation performance and cheese quality. Since the identification of the impact of milk protein polymorphisms on milk quality and its technological properties (Aschaffenburg and Drewry, 1955), milk protein variants have been intensively characterized in different species including goats (Marletta et al., 2007; Caroli et al., 2009; Giambra and Erhardt, 2012; Selvaggi et al., 2014; Ozdemir et al., 2018). As a side effect of the intensive work, simultaneous publications led to inconsistency in the naming and positioning of some sequence variants which were anchored in part to mature and in part to immature proteins and sometimes include mutations in non-coding regions. In addition, many studies have referred to the genetic and protein variants of caseins in goats with different nomenclature. This review gives a comprehensive overview on identified protein and DNA variants of the four casein genes in goats. One goal is to clarify names and nomenclatures, the positions of nucleotide and amino acid polymorphisms and provide a translation from DNA to protein variant. We focus on protein variants, but also describe linked nucleotide variants in non-coding regions of the gene, like the promoter, 5′ and 3’ untranslated regions, and introns, which could affect the transcription and translation and thereby production traits. Furthermore, we have derived haplotypes across all four linked genes and summarize the frequency of alleles in different breeds and the impact of milk protein variants on milk quality for cheese making, human health and human nutrition.

## CSN1S1

At the functional level, alpha-S1-casein is required for the efficient export of other caseins from the endoplasmic reticulum to the Golgi compartment, the casein transport in the secretory pathway, and for the biogenesis of casein micelles in milk (Chanat et al., 1999; Le Parc et al., 2010). Alpha-S1-casein has an important role in the capacity of milk to provide calcium phosphate for human nutrition (Kolb et al., 2011).

The percentage of α-S1-casein in goat milk varies from 0 to 25%. Alpha-S1-casein concentrations has been shown to be dependent on the existence of polymorphisms in the coding and regulatory sequences of the gene (Boulanger et al., 1984). In contrast, the α-S1-casein fraction in bovine milk constitutes up to 40% (Farrell et al., 2004). Strong, intermediate, low, and null expression levels of α-S1-casein in goat milk are distinguished (Moatsou et al., 2004; Caroli et al., 2007; Kupper et al., 2010). In the first group comprising CSN1S1*A, CSN1S1*A3, CSN1S1*B1, CSN1S1*B2, CSN1S1*B3, CSN1S1*B4, CSN1S1*C, CSN1S1*H, CSN1S1*L, and CSN1S1*M are “strong” variants, which are associated with a high content of α-S1-casein in milk. CSN1S1*E and CSN1S1*I are “intermediate” variants associated with a medium content. CSN1S1*D, CSN1S1*F, and CSN1S1*G are “weak” variants associated with a low content, and CSN1S1*N, CSN1S1*01, and CSN1S1*02 are “null” variants leading to the absence of α-S1-casein in the milk of animals homozygote for these alleles (Grosclaude et al., 1987; Brignon et al., 1990; Chianese et al., 1997; Grosclaude and Martin, 1997; Martin et al., 1999; Bevilacqua et al., 2002; Ramunno et al., 2005; Mestawet et al., 2013). The α-S1-casein strong variants produced around 3.6 g/L per allele, medium variants produced 1.1–1.6 g/L per allele, weak variants produced 0.45–0.6 g/L per allele, and null variants resulted in absence of the α-S1-casein in milk (Grosclaude et al., 1987; Grosclaude and Martin, 1997; Martin et al., 1999). Strong variants with high content of α-S1-casein are most desired in dairy production since they are associated with lower pH values, better coagulation properties, faster curdling rate and greater gel firmness, which contribute largely to high yield and quality of cheese, even if the rennet coagulation time was longer (Ambrosoli et al., 1988; Pirisi et al., 1994; Clark and Sherbon, 2000; Schmidely et al., 2002; Zullo et al., 2005; Caravaca et al., 2011; Devold et al., 2011; Cebo et al., 2012; Vacca et al., 2014; Johansson et al., 2015; Marković et al., 2018). Since the casein content in milk also affects the size of casein micelles and the amount and composition of milk fatty acids, cheese made from milk with strong α-S1-variants has a better structure and taste with less typical goat flavor than cheese produced from intermediate or weak variants (Delacroix Buchet et al., 1996). Generally, the lower α-S1-casein content in goat milk compared to bovine milk is associated with smaller casein micelles, with less hydrated pores (Park et al., 2007; Ingham et al., 2018; Nguyen et al., 2018). Hence, yoghurt (Miocinovic et al., 2016; Nguyen et al., 2018) and cheese (Park et al., 2007; Mestawet et al., 2014) made from goat milk have a lower dense gel structure and poor coagulation features compared to their counterparts made with cow milk.

The *CSN1S1* gene is located on chromosome 6 between 85.978 and 85.995 Mb and spans 16,808 bp. The DNA and protein reference sequences NC_030813 (goat genome reference version LWLT01 (Bickhart et al., 2017)) and XP_017904616 (NCBI database), respectively, represent the *CSN1S1* ^*^A protein variant (Boulanger et al., 1984; Brignon et al., 1989; Ramunno et al., 2004). The gene encodes 214 amino acids of α-S1-casein, the first 15 amino acids form the signal peptide sequence, followed by 199 amino acids long mature protein (Brignon et al., 1990). It consists of 19 exons ranging in size from 24 (exons 5, 6, 7, 8, 10, 13, 16) to 385 bp (exon 19) and 18 introns from 90 bp (intron 10) to 1,685 bp (intron 2) (Ramunno et al., 2004).

In the following, we briefly describe the 20 protein variants that have been identified so far (Supplementary Table S1 **)**.

The α-S1-casein CSNS1*A is the reference protein*.* The sub variants CSN1S1*A′ and CSN1S1*A2 represent the same reference protein, namely CSN1S1*A, though CSN1S1*A′ carries a T/C transition in intron 9 (Kupper et al., 2010) and CSN1S1*A2 a G/A transition in the 5′ flanking region (Ramunno et al., 2005). The same G/A transition is present also in variants CSN1S1*N and CSN1S1*01, which are null variants (Ramunno et al., 2005), which means absence of α-S1-casein in the milk of homozygous carriers. Hence, the G/A variation in the 5′ flanking region is not responsible for the absence of α-S1-casein in milk. Different from the other CSN1S1*A proteins, CSN1S1*A3 carries a transversion from A to C at position CHR6:85987198 (exon 10) leading to the amino acid exchange from Gln_77_ to Pro_77_ (Mestawet et al., 2013). CSN1S1*A is the predominant protein variant in most Indian breeds (frequency >0.7) (Kumar et al., 2007; Rout et al., 2010) and in a few breeds from Spain, Italy and France (frequency >0.5, Supplementary Table S2) (Ramunno et al., 1991).

The CSN1S1*B protein is divided into four variants (CSN1S1*B1, CSN1S1*B2, CSN1S1*B3, CSN1S1*B4) (Grosclaude and Martin, 1997). All CSN1S1*B variants differ from the reference protein through the substitution of Gln_77_ to Glu_77_ (Brignon et al., 1989), a substitution that is associated with the presence of a phosphate group on Serine at position 75 (Brignon et al., 1989). Among all α-S1-casein variants, CSN1S1*B1 is considered the ancestor allele showing the closest homology to its bovine and ovine counterparts (Grosclaude and Martin, 1997). CSN1S1*B1 and CSN1S1*B′ are identical proteins; the only difference is a synonymous SNP in the codon for Ser_66_ in exon 9 in CSN1S1*B′ (Caroli et al., 2007). The caseins CSN1S1*B2, CSN1S1*B3 and CSN1S1*B4 carry the additional T to C transition at position CHR6:85982615 (exon 4), leading to the substitution of Leu_16_ to Pro_16_ (Grosclaude and Martin, 1997; Cosenza et al., 2008). CSN1S1*B3 and CSN1S1*B4 carry the G to A transition at position CHR6:85988705 (exon 12) changing Arg_100_ to Lys_100_ (Grosclaude and Martin, 1997; Cosenza et al., 2008). CSN1S1*B4 differs from CSN1S1*B3 by the nucleotide substitution A to G at position CHR6:85993465 (exon 17) altering Thr_195_ to Ala_195_ (Grosclaude and Martin, 1997; Cosenza et al., 2008). With respect to names, it is necessary to mention that firstly, CSN1S1*B2 was named simply CSN1S1*B in old papers (Boulanger et al., 1984; Brignon et al., 1989), and secondly, the variant CSN1S1*E had also been named CSN1S1*B^−^ (Grosclaude et al., 1987). CSN1S1*B protein variants can be found at high frequency (>0.5) in diverse breeds across the world (Supplementary Table S2).

CSN1S1*C and CSN1S1*C1 differ from the reference by five and four amino acid substitutions, respectively. The two variants carry the amino acid substitutions His_8_ to Ile_8_, Leu_16_ to Pro_16_, Gln_77_ to Glu_77_, and Arg_100_ to Lys_100_. The additional substitution Thr_195_ to Ala_195_ occurs only in CSN1S1*C (Leroux et al., 1990; Rahmatalla et al., 2021).

CSN1S1*D is characterized by a deletion of 11 amino acids at positions 59 to 69 which comprises the major phosphorylation site of the protein (Brignon et al., 1990). The deletion of the single nucleotide C at position CHR6:85986426 in exon 9 is a splice-site mutation which causes the exclusion of exon nine in the mature RNA assembly which also reduces the synthesis rate of the protein (Mahé and Grosclaude, 1989; Brignon et al., 1990). Besides the significant deletion, CSN1S1*D carries the substitutions Leu_16_ to Pro_16_ and Gln_77_ to Glu_77_, which also occur in most CSN1S1*B and CSN1S1*C protein variants (Mahé and Grosclaude, 1989; Brignon et al., 1990).

CSN1S1*E (formerly CSN1S1*B′) has the same amino acid variant as CSN1S1*B4. Besides that, CSN1S1*E has an insertion of 457–458 bp in exon 19, which is expressed but untranslated (Jansàpérez et al., 1994). This insert is a truncated long interspersed repeated element (LINE), containing part of the ORF-2, the 3′ UTR, and the original retrotransposon’s poly (A) tail. The mutant 3′ UTR likely controls the mRNA stability of transcripts and/or the translation rate which was seen as low casein content in goat milk of CSN1S1*E carriers (Jansàpérez et al., 1994). The CSN1S1*E allele is highly frequent (>0.5) in Saanen goats worldwide (Tadlaoui Ouafi et al., 2002; Maga et al., 2009), and in other breeds from Spain, France, and the United States (Supplementary Table S2) (Grosclaude et al., 1994; Jordana et al., 1996; Tadlaoui Ouafi et al., 2002; Caravaca et al., 2008; Maga et al., 2009).

CSN1S1*F contains the amino acid variants Pro_16_ and Glu_77_ as in CSN1S1*D (Grosclaude et al., 1987; Brignon et al., 1990). Most significant in CSN1S1*F is the lacks of 37 amino acids from position 59 to 95 as the consequence of a frameshift mutation due to a single nucleotide deletion in exon 9 (Grosclaude et al., 1987; Brignon et al., 1990). The deletion starts exactly at the same position and with the same mutation as in CSN1S1*D. Two additional insertions of 11 and 3 base pairs in intron nine are likely responsible for the skipping of exons 9, 10 and 11 in CSN1S1*F (Leroux et al., 1992). The content of this shortened α-S1-casein protein variant in milk is low, likely as a result of low efficiency and accuracy of the splicing machinery (Martin et al., 1999), which might result from either a reduced translation rate or intracellular degradation of the incomplete protein. Several breeds that originate from the Alpine region carry the CSN1S1*F allele at a frequency above 0.40 (Grosclaude et al., 1987; Ramunno et al., 1991). A higher expression of CSN1S1*F compared to CSN1S1*N might be attributed to a mutation in the promotor region creating an extra putative activator protein 1 (AP-1) binding motif (Ramunno et al., 2005). However, experimental validation does not exist. Particular high frequency >0.90 of CSN1S1*F was found in Toggenburg goats from America and Orobica goats from Italy (Supplementary Table S2) (Caroli et al., 2006; Maga et al., 2009).

Protein variant CSN1S1*G is missing 13 amino acids from position 14 to 26. The G/A transition at position CHR6:85982647 in the 5′ splice site at the beginning of intron 4 leads to the skipping of exon 4 (Martin and Leroux, 1994). The occurrence of the CSN1S1*G allele is associated with low α-S1-casein content in milk (Martin and Leroux, 1994).

CSN1S1*H differs from the reference variant CSN1S1*A by a single amino acid substitution at position 1, Arg_1_ to Lys_1_. This protein was classified as a strong variant (Chianese et al., 1997). For the Italian Garganica breed the frequency of 0.07 was reported (Sacchi et al., 2005).

For the protein variants CSN1S1*G, CSN1S1*I, CSN1S1*J, CSN1S1*K, CSN1S1*L, and CSN1S1*M there is information available for sequence variants only, but not for allele frequency in any breed. CSN1S1*I had the same amino acids sequence as variant CSN1S1*A. However, CSN1S1*I had only about half of the amount of casein in the milk than CSN1S1*A (Chianese et al., 1997), which led to the differentiation between CSN1S1*I and CSN1S1*A. The causal mutation is unidentified.

CSN1S1*J carries the combination of the amino acids substitutions Gln_77_ to Glu_77_ and Arg_100_ to Lys_100_, which are also found in other protein variants (Rahmatalla et al., 2021). CSN1S1*J detected in Bezoar ibex. This variant is similar to CSN1S1*B1 which is consider the ancestor allele, but it contains the Arg_100_ to Lys_100_ substitution (Rahmatalla et al., 2021).

The CSN1S1*K protein variant differs from the reference by the unique amino acid substitution from Ile_44_ to Val_44_ as a results of a to G transition at position CHR6:85984154 (exon 7) (Rahmatalla et al., 2021). The CSN1S1*K was found in wild Alpine ibex (Rahmatalla et al., 2021).

CSN1S1*L harbors the two amino acids Pro_16_ and Glu_77_ instead of Leu_16_ and Gln_77_ in the reference sequence and additionally the amino acid substitution from Arg_90_ to His_90_ (Chianese et al., 1997). The transition of G to A at CHR6:85987327 in exon 11 leads to the substitution of Arg_90_ to His_90_.

CSN1S1*M carries the Leu_16_ to Pro_16_ substitution, which occurs also in other protein variants. CSN1S1*M also contains the amino acid substitution of Ser_66_ to Leu_66_ which results from a C/T transition at CHR6:85986426 in exon 9 (Chianese et al., 1998; Bevilacqua et al., 2002).

CSN1S1*N, CSN1S1*01 and CSN1S1*02 are null variants resulting in truncated gene products and subsequently no casein in the milk. CSN1S1*N descends from protein variant CSN1S1*M, but contains also the nucleotide deletion of a single C at CHR6:85986426 in exon 9 that also occurs in variants CSN1S1*D and CSN1S1*F, where it causes exon skipping. However, in CSN1S1*N the one-nucleotide deletion leads to a premature stop codon. The amount of the truncated transcript is reduced to one-third and the amount of α-S1-casein in milk in homozygous carriers is null (Ramunno et al., 2002; Ramunno et al., 2005). CSN1S1*01 is a truncated protein where the last seven exons are missing. The truncation results from a deletion of about 8.5 kb starting from nucleotide 181 of intron 12. In homozygous carriers, α-S1-casein is absent in milk (Grosclaude et al., 1987; Leroux et al., 1990; Cosenza et al., 2003). CSN1S1*02 is another null variant, leading to the absence of the respective protein in milk. Martin et al. (1999) proposed a large insertion that has not been identified so far. Albeit the goats carrying the null alleles homozygous do not have contain α-S1-casein in their milk, the Indian breeds Ganjam and Local MP have an allele frequencies of 0.40 and 0.45, respectively (Rout et al., 2010).

Goat milk that lacks α-S1-casein has a higher pH value and a low content of chemical components like total solids, protein, casein, Nitrogen, fat, and Phosphorus compared to milk with strong α-S1-casein variants (Ambrosoli et al., 1988; Pirisi et al., 1994; Clark and Sherbon, 2000; Devold et al., 2011; Johansson et al., 2015; Marković et al., 2018). In addition, milk containing α-S1-casein null variants is characterized by large casein micelles with weak gel firmness and long coagulation time (Devold et al., 2011; Johansson et al., 2015). The gross yield of cheese and the content of fat and non-fat solids as well as the fat to dry matter ratio and the total solids recovery rate from milk that lacks α-S1-casein is low (Pirisi et al., 1994). Furthermore, textural parameters of cheese such as hardness and plasticity were low in cheese that is produced from goat milk lacking α-S1-casein (Pirisi et al., 1994). In contrast, milk containing strong α-S1-casein variants produce firmer curd that is associated with high gross yield of cheese with a better composition (Devold et al., 2011; Johansson et al., 2015).

## CSN2

Beta-casein is the most hydrophobic casein as a result of a highly charged N-terminal domain and an anionic phosphoserine cluster (Swaisgood, 2003). Beta-casein is the major protein in goat milk (Clark and Mora Garcia, 2017) comprising up to 50% of the total casein content. In comparison, human milk casein contains 60%–70% and bovine milk casein 36% β-casein (Guo, 2009; Elagamy, 2016). Beta-casein is a primary source of essential amino acids and facilitates the mineral transport for infants (Sadler and Smith, 2013). The peptides derived from β-casein during digestion have antihypertensive and immune-stimulating properties (Silva et al., 2006; Lamothe et al., 2007).

The *CSN2* gene is located on chromosome 6 between 86.006 and 86.015 Mb (NC_030813, goat genome reference version LWLT01) (Bickhart et al., 2017). The 9,072 bp long gene consists of nine exons ranging in size from 24 (exon 5) to 492 bp (exon 7) (Roberts et al., 1992; Hayes and Petit, 1993). The protein contains 222 amino acids, which comprise 207 functional amino acids and 15 amino acids of a signal peptide (XP_005681778 (NCBI database).

So far, eight protein variants have been identified (Table 1). Variants CSN2*A, CSN2*B, CSN2*C, CSN2*D, and CSN2*E are “strong” variants associated with high β-casein content in milk (5 g/L per allele) (Roberts et al., 1992; Mahé and Grosclaude, 1993; Neveu et al., 2002; Galliano et al., 2004; Cosenza et al., 2005; Caroli et al., 2006). The variants CSN2*C2 and CSN2*F1 were associated with intermediate β-casein content (3.3 and 2.7 g/L per allele, respectively) (Nicolai et al., 2021). The two null alleles CSN2*0 and CSN2*0′ are associated with a lack of β-casein in milk (Persuy et al., 1999). In the *CSN2* gene, the existence of the strong variants were associated with the highest milk protein content and yield in Sarda goats from Italy (Vacca et al., 2014).

**TABLE 1:** Nucleotide and amino acid variants within the gene and mature protein of the *CSN2*.

Encoded protein variant	Protein/DNA variant name	Position on chr 6 (bp)^a^	86,008,407	86,008,401	86,008,103	86,008,049	86,008,047	86,008,016	86,007,341/86,007,342	86,006,394	References
		Position on the gene	Exon 7	Exon 7	Exon 7	Exon 7	Exon 7	Exon 7	Exon 8	Exon 9 (3′ UTR)	
		Amino acid position^b^	47	58	148	166	167	177	207			
CSN2*C	CSN2*C	NC_030813	C	T	G	G	G	A	AC	G	Wang et al. (2001); Neveu et al. (2002); Bickhart et al. (2017)
		XP_005681778	Asp	Leu	Pro	Ser	Q	Val	Val		
CSN2*A	CSN2*A	CSN2*A_Gen.						G			Roberts et al. (1992), Rando (1998)
		CSN2*A_Prot.						Ala			
CSN2*A	CSN2*A1	CSN2*A1_Gen.								***A***	Cosenza et al. (2005)
		CSN2*A1_Prot.									
CSN2*B	CSN2*B*	CSN2*B_Gen.									NCD; Mahé and Grosclaude (1993)
		CSN2*B_Prot.									
CSN2*C	CSN2*C1	CSN2*C1_Gen.								***A***	Chessa et al. (2008)
		CSN2*C1_Prot.									
CSN2*C	CSN2*C2**	CSN2*C2_Gen.								***A***	Nicolai et al. (2021)
		CSN2*C2_Prot.									
CSN2*D	CSN2*D	CSN2*D_Gen.							TT		NCD; Galliano et al. (2004)
		CSN2*D_Prot.							Asn		
CSN2*E	CSN2*E	CSN2*E_Gen.				T					Caroli et al. (2006)
		CSN2*E_Prot.				Tyr					
CSN2*F	CSN2*F (CSN2*E)	CSN2*F_Gen.	A								NCD; Chianese et al. (2007); Moatsou et al. (2007)
		CSN2*F_Prot.	Thr								
CSN2*F	CSN2*F1	CSN2*F1_Gen.	A							***A***	Nicolai et al. (2021)
		CSN2*F1_Prot.	Thr								
CSN2*G	CSN2*G (CSN2*F)	CSN2*G_Gen.			A						Rahmatalla et al. (2021)
		CSN2*G_Prot.			Leu						
CSN2*0	CSN2*0	CSN2*0_Gen.		Del_T							Persuy et al. (1999)
		CSN2*0_Prot.		Stop codon	NT	NT	NT	NT	NT		
CSN2*0	CSN2*0'	CSN2*0'_Gen.					A				Rando et al. (1996), Boulanger et al. (1984)
		CSN2*0'_Prot.					Stop codon^c^	NT	NT		

^a^ Chromosomal position in base pairs (bp) in the positive strand according to the goat genome reference version LWLT01, which represent CSN2*C.

^b^ Amino acids position according to the reference protein sequence XP_005681778. The whole sequence of 257 amino acids comprises 207 of the mature protein. All the amino acid position based on the mature protein.

*: Variant CSN2*B migrates faster under alkaline pH and slower in acid pH and there is difference in mobility compared to variant CSN2*A

**: CSN2*C2 coding for the same amino acid sequence as variant CSN2*C1, but it was lower the amount of the expressed protein.

^c^ The nucleotide mutation generates a stop codon.

Mutations in the 3'UTR are shown in italics and bold and non-synonymous mutations are shown in normal font.

Del= deletion, NCD = Not characterized at the DNA level.

NT: Not translated after stop codon.

The name of the old variant are given in parentheses.

The reference protein is variant CSN2*C (Wang et al., 2001; Neveu et al., 2002). CSN2*A differs from the reference by the amino acid substitution of Val_177_ to Ala_177_ (Neveu et al., 2002) as a result of an A/G transition at position CHR6:86008016 in exon 7 (Roberts et al., 1992). CSN2*A is considered the ancestral allele in goats because the amino acid alanine at position 177 is also found in sheep, cattle and buffalo (Chessa et al., 2005). The variant CSN2*A1 differs from CSN2*A by an additional G/A SNP in exon 9, which belongs to the 3′ untranslated region (Cosenza et al., 2005). CSN2*A was predominant with a frequency above 0.90 in Girgentana and Argentata dell’Etna from Italy, and in most Indian goats (Supplementary Table S3) (Marletta et al., 2005; Rout et al., 2010). A high frequency above 0.70 was evident in French Creole goats, West African breeds, and Buren goat from Germany (Supplementary Table S3) (Mahé and Grosclaude, 1993; Caroli et al., 2007; Kupper et al., 2010).

The CSN2*B variant was characterized *via* isoelectric protein electrophoresis, where CSN2*B migrates faster than CSN2*A (Mahé and Grosclaude, 1993). The mobility differences between CSN2*A and CSN2*B might be due to an extra phosphate group in CSN2*B (Mahé and Grosclaude, 1993).

CSN2*C1 and CSN2*C2 encode the same protein CSN2*C, the reference protein sequence. The protein variant CSN2*C is predominant with a frequency of ≥0.70 in most Italian goats, Angora and Hair goats from Turkey, Rajasthan and South goats from India as well as Banat White goats from Romania (Supplementary Table S3) (Chessa et al., 2005; Caroli et al., 2006; Chessa et al., 2007; Kusza et al., 2016). The two subtypes differ in a synonymous transition of G to A in the 3′ untranslated region of exon 9 in CSN2*C (Chessa et al., 2008; Nicolai et al., 2021). This single nucleotide polymorphism likely leads to lower amount of ß-casein if the animals is homozygouse for the CSN2*C2 subtype compared to CSN2*C1. An impact on the mRNA stability and or translation rate might be causal for the low amount of the protein (Nicolai et al., 2021). The variant CSN2*C1 was found with a frequency of more than 0.10 in Bunte Deutsche Edelziege and Weiße Deutsche Edelziege goats from Germany and Italian Girgentana goats (Kupper et al., 2010; Tortorici et al., 2014).

CSN2*D differs from the reference protein in the amino acid substitution from Val_207_ to Asn_207_ (Galliano et al., 2004). DNA sequence comparisons show a AC/TT substitution at positions CHR6:86007341 and CHR6:86007342 in exon 8 that is responsible for the amino acid change.

CSN2*E is characterized by the amino acid exchange from Ser_166_ to Thr_166_ which results from the G/T transversion at position CHR6:86008049 in exon 7 (Caroli et al., 2006). CSN2*E was found at a low frequency (0.08) only in Italian Frisa goats (Caroli et al., 2006).

The protein variant CSN2*F differs from the reference by the substitution of Asp_47_ to Thr_47_ (Chianese et al., 2007; Moatsou et al., 2007). The underlying SNP is a C/A transversion at position CHR6:86008407 in exon 7. By mistake, this protein variant had been named CSN2*E by Caroli et al. (2006) although CSN2*E already existed. It was correctly renamed as CSN2*F by Martin et al. (2013). CSN2*F1 and CSN2*F represent the same β-casein protein. CSN2*F1 differs from CSN2*F by a synonymous transition G/A in the 3′ untranslated region in exon 9 (Nicolai et al., 2021).

CSN2*G (previously CSN2*F) is characterized by the amino acid substitution from Pro_148_ to Leu_148_ and found in Wild Alpine ibex (Rahmatalla et al., 2021). The nucleotide mutation is a G/A substitution at position CHR6:86008103 in exon 7 (Rahmatalla et al., 2021).

Observations of milk lacking β-casein led to the conclusion that at least two null alleles exist (Dall’Olio et al., 1989; Mahé and Grosclaude, 1989). Variant CSN2*0 has one nucleotide deletion (T/-) at position CHR6:86008401 in exon 7 which introduces a premature stop codon at position 58 in the mature protein (Persuy et al., 1999). The CSN2*0 null variant was detected in a frequency below 0.10 in Creole goats from France and some Italian and Indian goats (Supplementary Table S3) (Mahé and Grosclaude, 1993; Marletta et al., 2005; Sacchi et al., 2005; Rout et al., 2010; Vacca et al., 2014). However, the frequency was 0.19 in Italian Garganica goats (Albenzio et al., 2009). CSN2*0′ carries the single nucleotide substitution C to T at position CHR6:86008047 in exon 7, which introduces a premature stop codon at position 167 of the mature protein (Rando et al., 1996). The CSN2*0′ null variant was found at a frequency below 0.10 in some Italian breeds and in Arbi goats from Tunis (Supplementary Table S3) (Chessa et al., 2005; Gigli et al., 2008; Vacca et al., 2009a; Tortorici et al., 2014). In CSN2*0′, another SNP in the promoter region was detected which negatively affects the transcriptional activity of the gene (Cosenza et al., 2007; Cosenza et al., 2016).

No information was available for the allele frequency of CSN2*C2, CSN2*D, CSN2*F, CSN2*F1, and CSN2*G.

The β-caseins in milk are primarily classified into the two types A1 and A2. A1 and A2 differ in the generation of β-casomorphin-7 (BCM-7) peptides during digestion (Jianqin et al., 2016). The peptide β-casomorphin-7 gained attention because it was associated with gastrointestinal disturbances, ischemic heart disease, arteriosclerosis, type 1 diabetes, and sudden infant death syndrome in humans (Birgisdottir et al., 2002; Tailford et al., 2003; Ho et al., 2014). The peptide β-casomorphin-7 is uniquely derived from the digestion of β-casein type A1, but not from A2. Different from cow milk, goat milk mainly contains the β-casein type A2 (Cieslinska et al., 2012). Therefore, β-casein of the type A2 contributes to the healthy properties of goat milk because it does not produce the peptide β-casomorphin-7 during digestion (De Noni and Cattaneo, 2010).

It is worth noting that with the increasing awareness of consumers’ behavior and their interest in healthy eating and functional food, different companies launched β-casein type A2 milk from cattle on the market. Costs are associated with the separate handling of this type of milk during processing. Since goat milk naturally contains β-casein type A2, this milk plays a crucial role in providing functional milk and milk products to the food market. A positive benefit for human health is realized here without additional marketing costs.

## CSN1S2

The alpha-S2-casein protein plays an important role in the transport of calcium phosphate. The alpha-S2-casein content in goat milk is relatively high comprising about 19%, while it constitutes up to 10% in bovine milk (McMahon and Brown, 1984). The antimicrobial peptide cathelicidin-1 which derives from the digestion of alpha-S2-casein has the activity to inhibit the growth of Gram-positive and Gram-negative bacteria (Zucht et al., 1995; López-Expósito et al., 2008; Gigli, 2016).

The *CSN1S2* gene resides on chromosome 6 between 86.077 and 86.094 Mb, which is about 16,695 bp long and consists of 18 exons (NCBI, accession no. NC_030813). The gene encodes 223 amino acids comprising 208 functional amino acids and 15 amino acids of a signal peptide (https://www.uniprot.org/uniprot/P33049).

The DNA [NC_030813, goat genome reference version LWLT01 (Bickhart et al., 2017)] and protein reference sequence (XP_013820127.2, NCBI database) represent the CSN1S2*A variant (Boulanger et al., 1984; Bouniol et al., 1993; Bickhart et al., 2017). CSN1S2*A is considered the ancestor variant for goat according to the evolutionary path from sheep and cattle (Sacchi et al., 2005). This variant is common with a frequency of ≥0.70 in distinct Saanen goats, most Italian, German and Indian goats, Dwarf goats from Nigeria, and Nubian goats from Sudan (Supplementary Table S4) (Boulanger et al., 1984; Bouniol et al., 1994; Ramunno et al., 2001b; Erhardt et al., 2002; Marletta et al., 2004; Chiatti et al., 2005; Marletta et al., 2005; Caroli et al., 2007; Chessa et al., 2007; Kupper et al., 2010; Rout et al., 2010; Yue et al., 2013).

So far, 14 alpha-S2-casein protein variants have been identified (CSN1S2*A, CSN1S2*B, CSN1S2*C, CSN1S2*D, CSN1S2*E, CSN1S2*F, CSN1S2*G, CSN1S2*H, CSN1S2*I, CSN1S2*J, CSN1S2*K, CSN1S2*0, truncated sub-variant A, and truncated sub-variant E) (Table 2) (Boulanger et al., 1984; Bouniol, 1993; Bouniol et al., 1994; Veltri et al., 2000; Ramunno et al., 2001a; Ramunno et al., 2001b; Lagonigro et al., 2001; Erhardt et al., 2002; Cunsolo et al., 2006; Rahmatalla et al., 2021). While the protein variants CSN1S2*A, CSN1S2*B, CSN1S2*C, CSN1S2*E, CSN1S2*F, and CSN1S2*G are associated with normal amounts of α-S2-casein of about 2.5 g/L per allele (Boulanger et al., 1984; Grosclaude et al., 1987; Bouniol et al., 1994; Ramunno et al., 2001a), CSN1S2*D is associated with a reduced amount of about 1.5 g/L per allele (Ramunno et al., 2001a). In CSN1S2*0 homozygous goats, alpha-S2-casein was not detectable (Ramunno et al., 2001b).

**TABLE 2:** Nucleotide and amino acid variants within the gene and mature protein of the *CSN1S2*.

Encoded protein variant	Protein/DNA variant name	Position on chr 6 (bp)^a^	86,080,969	86,081,790	86,081,847	86,081,887	86,084,638	86,085,134	86,085,160	86,085,167	86,085,714	86,089,401	86,089,407	86,089,479	References
		Position on the gene	E–3	E–4	I–4	E–5	E–9	E–11	E–11	E–11 & I–12	E–12	E–16	E–16	E–16	
		Amino acid position^b^	7	17		20	64	110	119	121-122-123-124	127	167	169	197	
CSN1S2*A	CSN1S2*A	NC_030813	G	T		T	G	G	G		G	A	G	C	Boulanger et al. (1984); Bouniol et al. (1993); Bickhart et al., 2017
		XP_013820127.2	Val	Phe		Ile	Glu	W	Ala	Thr-Pro-Thr-Val	Glu	Lys	Ser	Pro	
CSN1S2*B	CSN1S2*B	CSN1S2*B_Gen.					A								Boulanger et al. (1984); Bouniol et al. (1993)
		CSN1S2*B_Prot.					Lys								
CSN1S2*C	CSN1S2*C	CSN1S2*C_Gen.										T			Bouniol et al. (1994)
		CSN1S2*C_Prot.										Ile			
CSN1S2*D	CSN1S2*D	CSN1S2*D_Gen.								Del-1					Ramunno et al. (2001a)
		CSN1S2*D_Prot.								Asn-Del-2-Del-3-Del-4					
CSN1S2*E	CSN1S2*E	CSN1S2*E_Gen.			Del-5							T		G	Veltri et al. (2000); Lagonigro et al. (2001)
		CSN1S2*E_Prot.										Ile		Arg	
CSN1S2*F	CSN1S2*F	CSN1S2*F_Gen.	A												Ramunno et al. (2001a)
		CSN1S2*F_Prot.	Ile												
CSN1S2*G	CSN1S2*G*	CSN1S2*G_Gen.													NCD; Erhardt et al. (2002)
		CSN1S2*G_Prot.													
CSN1S2*H	CSN1S2*H	CSN1S2*H_Gen.							C						Rahmatalla et al. (2021)
		CSN1S2*H_Prot.							Pro						
CSN1S2*I	CSN1S2*I	CSN1S2*I_Gen.							C		A				Rahmatalla et al. (2021)
		CSN1S2*I_Prot.							Pro		Lys				
CSN1S2*J	CSN1S2*J	CSN1S2*J_Gen.				C			C				A		Rahmatalla et al. (2021)
		CSN1S2*J_Prot.				Thr			Pro				Asn		
CSN1S2*K	CSN1S2*K	CSN1S2*k_Gen.		C		C			C		A		A		Rahmatalla et al. (2021)
		CSN1S2*K_Prot.		Ser		Thr			Pro		Lys		Asn		
CSN1S2*0	CSN1S2*0	CSN1S2*0_Gen.						A							Ramunno et al. (2001b)
		CSN1S2*0_Prot.						Pre mature Stop codon	NT	NT	NT	NT	NT	NT	
*Truncated sub variant A*	Truncated sub variant A^c^	Truncated sub variant A_Gen.													NCD; Cunsolo et al., 2006
		Truncated sub variant A_Prot.													
*Truncated sub variant E*	Truncated sub variant E^c^	Truncated sub variant E_Gen.													NCD; Cunsolo et al., 2006
		Truncated sub variant E_Prot.													

^a^ Chromosomal position in base pairs (bp) in the positive strand according to the goat genome reference version LWLT01, which represent CSN1S2*A.

^b^ Amino acids position according to the reference protein sequence XP_013820127.2. The whole sequence of 223 amino acids comprises 208 of the mature protein. All the amino acid position based on the mature protein.

*: CSN1S2*G variant has an isoelectric point between CSN1S2*A and CSN1S2*C.

Del-1: Deletion of 106 bp (CTCCCACCGTGGTGAGTGCTGCTTTTTTATATGTTGTTTCTTGTTTATTTTTTTTTTCTTTCTGTTTTTGGTGAAGGATGAGTTCAGGATAAAGATTTGTAAAATG)

^c^ Deletion of the C-terminal tetra peptide.

Del-2: Deletion of the amino acid Proline (Pro).

Del-3: Deletion of the amino acid Threonine (Thr).

Del-4: Deletion of the amino acid Valine (Val).

Del-5: Deletion of 4 bp (AAAT).

Del.: Deletion.

NCD: Not characterize at the DNA level.

NT: Not translated after stop codon.

CSN1S2*B differs from the reference (CSN1S2*A) by a G/A substitution at CHR6:86084638 in exon 9, which is responsible for the amino acid substitution Glu_64_ to Lys_64_ that affects a phosphorylation site (Boulanger et al., 1984; Bouniol et al., 1994). CSN1S2*B occurs at a frequency above 0.40 in Egyptian and Lithuanian Native goats (Othman and Ahmed, 2006; Baltrėnaitė et al., 2009).

CSN1S2*C distinguishes from the reference by the transversion A/T at CHR6:86089401 in exon 16 which changing Lys_167_ to Ile_167_ (Bouniol et al., 1994). This variant occurs at a frequency ≥0.40 in Vallesana and Roccaverano Italian goats, Dwarf Cameroon goats, Borno goats from Nigeria, Hair goats from Turkey, as well as Maharashtra and Rajasthan from India (Supplementary Table S4) (Sacchi et al., 2005; Caroli et al., 2007; Chessa et al., 2007).

CSN1S2*D contains a 106 bp deletion comprising the last 11 bp of exon 11 and the first 95 bp of the following intron (Ramunno et al., 2001a). This deletion is responsible for the lack of the three codons Pro_122_, Thr_123_, and Val_124_. Furthermore, the last undeleted nucleotide (A) of exon 11 together with the two nucleotides (AT) of the following intron generate a codon for the amino acid Asn121 instead of Thr121.which is followed by a new GT dinucleotide splicing donor site. The rare CSN1S2*D variant was found in Italian Argentata dell’Etna and Girgentana goats and Hungarian goats with a frequency of 0.01 (Kusza et al., 2007; Gigli et al., 2008).

CSN1S2*E is characterized by a C/G mutation at CHR6: 86089479 in exon 16, leading to the amino acid exchange Pro_197_ to Arg_197_ (Chianese et al., 1998; Veltri et al., 2000; Lagonigro et al., 2001). Additionally, the of A/T transversion of CSN1S2*C changing Lys_167_ to Ile_167_ is present also in CSN1S2*E. Furthermore, a deletion of 4 bp (AAAT) was identified in intron 4 (Lagonigro et al., 2001). CSN1S2*E was found at low frequency (below 0.11) in some Italian goats, Hair goats from Turkey, and South goats from India (Sacchi et al., 2005; Caroli et al., 2006; Chessa et al., 2007; Gigli et al., 2008; Albenzio et al., 2009; Vacca et al., 2009b; Palmeri et al., 2014; Vacca et al., 2014). In addition to the full-length protein variants CSN1S2*A and CSN1S2*E, two truncated sub-variants were identified, which lack the C-terminal tetrapeptide (Gln_199_, Pro_200_, Leu_201_, Gln_202_, Pro_203_, Leu_204_). These truncated variants likely originate from differential splicing of pre-messenger RNA during the translation process of CSN1S2*A and CSN1S2*E variants (Cunsolo et al., 2006).

CSN1S2*F differs from the reference by a G/A transition at CHR6:86080969 in exon 3 substituting Val_7_ to Ile_7_ (Ramunno et al., 2001a). CSN1S2*F is common (frequency ≥0.40) in Italian Garganica, Orobica, Frisa, Sarda goats, and Weiße Deutsche Edelziege (Sacchi et al., 2005; Caroli et al., 2006; Vacca et al., 2009b; Kupper et al., 2010).

There is not much known about the nucleotide and protein sequence of the variant CSN1S2*G. This protein has an isoelectric point between CSN1S2*A and CSN1S2*C (Erhardt et al., 2002).

CSN1S2*H differs from CSN1S2*A by a G/C substitution at CHR6:86085160 in exon 11 leading to the substitution of Ala_119_ by Pro_119_ (Rahmatalla et al., 2021). This variant is predominant in Sudanese goats (Supplementary Table S4) (Rahmatalla et al., 2021).

CSN1S2*I contains the same mutations that distinguish CSN1S2*G from the reference and additionally a G/A substitution at CHR6:86085714 in exon 12 that is responsible for the change of Glu_127_ to Lys_127_ (Rahmatalla et al., 2021). So far, CSN1S2*I has been detected in Nubian and Desert goats only at a frequency of 0.21 and 0.10, respectively (Rahmatalla et al., 2021).

CSN1S2*J diverges from the reference by a T/C substitution at CHR6:86081887 in exon 5 and G/A substitution at CHR6:86089407 in exon 16 that account for the substitutions Ile_20_ to Thr_20_ and Ser_169_ to Asn_169_, respectively (Rahmatalla et al., 2021). In addition, the mutation which distinguishes CSN1S2*H from CSN1S2*A is present also in CSN1S2*J (Rahmatalla et al., 2021).

CSN1S2*K carries the same three amino acid substitution that occur in CSN1S2*J (Rahmatalla et al., 2021) and additionally the amino acid substitutions Phe_17_ to Ser_17_ and Glu_127_ to Lys_127_ as a result of the nucleotide substitutions of T to C at CHR6:86081790 in exon 4 and G to A at CHR6:86085714 in exon 12, respectively (Rahmatalla et al., 2021) The CSN1S2*J and CSN1S2*K were identified in wild Nubian ibex (Rahmatalla et al., 2021).

CSN1S2*0 carries a stop codon at the amino acid position 110 as a result of a G to A transition at CHR6:86085134 in exon 11, which disrupts the codon for Try_110_ (Ramunno et al., 2001b). As a consequence, a truncated non-functional protein of 109 instead of 208 amino acids is generated. Homozygous CSN1S2*0 carriers do not have α-S2-casein in milk. Nevertheless, the allele frequency in the Indian breeds Ganjam and Local MP is 0.55 and 0.48, respectively (Rout et al., 2010).

No information was available for the allele frequency of CSN1S2*J, CSN1S2*K, and the truncated sub-variants of CSN1S2*A and CSN1S2*E.

*CSN1S2* variants affected the milk protein content and milk yield in Sarda goats from Italy (Vacca et al., 2014). In Sarda goats, CSN1S2*AC genotyped animals produce the highest fat and protein yield, which affect the technological properties for cheese processing, while animals carrying the CSN1S2*CF genotype have the highest daily milk yield (Vacca et al., 2014). Interestingly, the variants CSN1S2*A, CSN1S2*C, and CSN1S2*F were associated with a high amount of α-S2-casein in milk and produced 2.5 g/l per allele (Boulanger et al., 1984; Bouniol et al., 1994; Ramunno et al., 2001a). In Chinese dairy goats, animals carrying the CSN1S2*FF genotype have higher milk fat and total solids content than CSN1S2*AA and CSN1S2*AF genotypes, but do not have lower milk yield (Yue et al., 2013). However, Lan *et al.* (2005) found significant lower milk yield of Xinong Saanen goats carrying the CSN1S2*FF genotypes compared to other genotypes. The contradictory effects of CSN1S2*FF genotyped animals might be attributed to the small sample size in the study of Lan et al. (2005). The variation in the α-S2-casein level in milk is associated with the unique physicochemical characteristic of milk. Thereby, α-S2-casein levels affect the cheese-making properties (Selvaggi and Tufarelli, 2011).

## CSN3

Kappa-casein (k-casein) is a calcium insensitive protein that forms a protective layer around the calcium-sensitive caseins α-S1, α-S2, and β, resulting in stable casein micelles (Quaglia and Gennaro, 2003). The formation, stabilization, and aggregation of casein micelles affect the physical characteristics and technological properties of milk (Alexander et al., 1988; Glantz et al., 2010). Kappa-casein is the only casein that is highly glycosylated (Haratifar and Guri, 2017). The mature kappa-casein protein has a labile peptide bond whose cleavage by chymosin or rennin produces a soluble hydrophilic glycopeptide called caseino-macropeptide (amino acids 106–171) and an insoluble peptide or para-k-casein (amino acids 1–105) (Yahyaoui et al., 2003). The caseino-macropeptide is important for milk coagulation.

Kappa-casein comprises about 20% in goat and sheep milk, in comparison, only 12–14% in cow milk (Fox and Brodkorb, 2008; Molik et al., 2012). Kappa-casein is essential for lactation. For example, mice lacking this gene (*CSN3* ^−/−^) fail to lactate because of destabilization of the micelles in the lumen of the mammary gland (Shekar et al., 2006). Kappa-casein contributes to the nutritional properties of milk. The protein has beneficial effects on oral health (Vacca-Smith and Bowen, 1995; Johansson and Lif Holgerson, 2011) and the digestion of k-casein generates an antimicrobial peptide (Malkoski et al., 2001).

The *CSN3* gene is located on chromosome 6 between 86.197 and 86.211 Mb [NC_030813, goat genome reference version LWLT01 (Bickhart et al., 2017)]. The gene spans 14,114 bp and comprises five exons (Martin et al., 2002). The protein contains 192 amino acids which include 21 amino acids of the signal peptide and 171 amino acids of the mature protein (Mercier et al., 1976; Coll et al., 1993). The mature protein is encoded in only two exons, exons 3 (9 amino acids) and exon 4 (162 amino acids) (Yahyaoui et al., 2003).

The *CSN3* gene in goats is highly polymorphic. To date, 35 variants are known that yield 24 protein variants and 11 synonymous mutations which are detectable only at the DNA level (Supplementary Table S5). In this review we follow the new nomenclatures of the k-casein variants recently described by Gautam et al. (2019). The DNA (NC_030813, goat genome reference version LWLT01 (Bickhart et al., 2017)) and protein reference sequences of *CSN3* (NP_001272516, NCBI database) represent the protein variant CSN3*B as the reference (Yahyaoui et al., 2001; Jann et al., 2004). We supplement the information with nucleotide positions in the coding sequence that is given in the reference X60763 (NCBI database) (Supplementary Table S5) (Coll et al., 1993) to facilitate the comparison between the position in the new goat reference genome of k-casein variants with previous studies that used the position in the coding sequence to name variants.

The reference protein CSN3*B is the predominant k-casein variant that occurs at a frequency >0.70 in most Italian and East Africa goats, Toggenberg and Buren goats from Germany, and Polish Saanen, fawn and white improved goats (Supplementary Table S6) (Chessa et al., 2003; Yahyaoui et al., 2003; Sacchi et al., 2005; Caroli et al., 2006; Kiplagat et al., 2010; Kupper et al., 2010; Strzelec and Niżnikowski, 2011; Rahmatalla et al., 2021).

The difference between CSN3*A and CSN3*B is the amino acid substitution Ile_119_ to Val_119_ which is the result of the transition of A to G at CHR6:86209184 in exon 4 (Coll et al., 1993; Yahyaoui et al., 2001). CSN3*A occurs also at a frequency ≥0.7 in Ionica and Montefalcone from Italy, Bunte and Weise Deutsche Edelziege, and Thueringer Waldziege from Germany as well as Ardi and Syrian goats from Saudi Arabia (Supplementary Table S6) (Caroli et al., 2001; Angiolillo et al., 2002; Kumar et al., 2009; El-Shazly et al., 2017). CSN3*A′ and CN3*A″ encode the same protein variant CSN3*A, but, carry the synonymous mutations T to C at CHR6:86209207 and C to T at CHR6:86209258 in exon 4, respectively (Kiplagat et al., 2010; Gautam et al., 2019). CSN3*A″ was found at low frequency in goats from Kenya and Maasai from Ethiopia. In that study, CSN3*A″ was named CSN3*O (Kiplagat et al., 2010).

CSN3*B′, CSN3*B″, CSN3*B‴ have the equivalent CSN3*B protein variant. In addition to the transition of G to A at CHR6:86209184 that alters the amino acid Val_119_ to Ile_119_, CSN3*B′, CSN3*B″, CSN3*B″ carry the synonymous mutations C to T at CHR6:86208883, C to T at CHR6:86209003, and A to G at CHR6:86209297 in exon 4, respectively (Supplementary Table S5) (Chessa and Bolla, 2003; Yahyaoui et al., 2003; Jann et al., 2004; Prinzenberg et al., 2005; Kiplagat et al., 2010). Previously, CSN3*B, CSN3*B′, CSN3*B″, and CSN3*B‴ had the names CSN3*D, CSN3*D′, CSN3*D″, and CSN3*P, respectively (Jann et al., 2004; Kiplagat et al., 2010). The homozygous CSN3*B genotype was associated with a higher milk protein content in Saanen goats (Catota-Gómez et al., 2017) and longer milk coagulation time in Spanish Murciano-Granadina goats compared to CSN3*A carriers (Caravaca et al., 2011).

CSN3*C differs from the reference variant CSN3*B in the three amino acid substitutions Val_65_ to Ile_65_, Ala_156_ to Val_156_, and Ser_159_ to Pro_159_ (Yahyaoui et al., 2001). The amino acid substitutions occurred as a result of a G to A, C to T, and T to C transitions at position CHR6:86209022, CHR6:86209296, and CHR6:86209304 in exon 4 (Yahyaoui et al., 2001), respectively. CSN3*C occurs at a frequency below 0.20 in some breeds from Italy, Canaria Spanish goats, goats from Germany and Turkey, Saanen goats worldwide, and Lithuania goats (Supplementary Table S6) (Chessa et al., 2003; Yahyaoui et al., 2003; Prinzenberg et al., 2005; Sacchi et al., 2005; Caroli et al., 2006; Gigli et al., 2008; Baltrėnaitė et al., 2009; Vacca et al., 2014; Catota-Gómez et al., 2017). CSN3*C′ differs from CSN3*C through a synonymous mutation in codon 131 (Prinzenberg et al., 2005).

In the CSN3*D (previously named CSN3*B), the amino acid substitutions Val_65_ to Ile_65_ and Ser_159_ to Pro_159_ that exist in CSN3*C were also found. In addition, CSN3*D carries the Gln_44_ to Arg_44_ substitution because of an A to G transition at CHR6:86208960 in exon 4 (Caroli et al., 2001; Yahyaoui et al., 2001). This variant occurs at a frequency of less than 0.40 in Italian and German goats and Ardi goats from Saudi Arabia (Supplementary Table S6).

CSN3*D′ differs from CSN3*D by a synonymous mutation in codon 56 and occurs at a frequency of 0.01 in Girgentana goats from Italy (Gerlando et al., 2015).

The CSN3*E protein variant differs from the reference by the amino acid substitution of Asp_90_ by Gly_90_ as the results of an A to G transition at position CHR6:86209098 in exon 4 (Angiolillo et al., 2002). CSN3*E occurs in Montefalcone goats from Italy at a frequency up to 0.45 (Angiolillo et al., 2002; Yahyaoui et al., 2003).

CSN3*F differs from the reference protein in the amino acid substitution of Ser_159_ by Pro_159_ which occurred due to the T to C transition at CHR6:86209304 in exon 4 (Yahyaoui et al., 2003). This variant was detected in breeds from Italy at a frequency below 0.15 and in Wild Spanish at a frequency of 0.98 (Yahyaoui et al., 2003). The variants CSN3*F′ and CSN3*F encode the same CSN3*F protein. CSN3*F differs from CSN3*F′ by a synonymous mutation in codon 77. CSN3*F′ occurs at a low frequency (0.01) in Raigarh goats from India (Gautam et al., 2019).

CSN3*G is characterized by G to A and T to C mutations at CHR6:86209022, and CHR6:86209304 in exon 4, respectively, in comparison to the reference variant CSN3*B. The mutations cause the amino acid exchange Val_65_ to Ile_65_ and Ser_159_ to Pro_159_, respectively (Yahyaoui et al., 2003). An addition synonymous mutation is present in codon 43 (Yahyaoui et al., 2003). CSN3*G was found at a frequency of less than 0.15 in a few breeds from Italy and Hair and Angora goats from Turkey (Yahyaoui et al., 2003; Prinzenberg et al., 2005; Gigli et al., 2008; Gerlando et al., 2015). Variant CSN3*G′ has the same amino acid substitutions as variant CSN3*G, but does not contain the synonymous mutation in codon 43 (Supplementary Table S5). The name CSN3*G′ was introduced by Gautam et al. (2019); before that this variant had been named CSN3*F or CSN3*L (Jann et al., 2004; Kiplagat et al., 2010). CSN3*G″ contains the same amino acid substitution as variant CSN3*G and in addition two synonymous mutations in codon 43 and 56 (Vacca et al., 2014; Gerlando et al., 2015; Gautam et al., 2019). The name CSN3*G″ was also introduced by Gautam et al. (2019) before this variant had been named CSN3*N and CSN3*S (Vacca et al., 2014; Gerlando et al., 2015).

CSN3*H differs from CSN3*B in the amino acid substitution from Asn_53_ to Ser_53_ due to the transition of A to G in exon 4 at CHR6:86208987 (Jann et al., 2004).

CSN3*I varies from the reference in the amino acid substitution Val_65_ to Ile_65_ as a result of a G to A transition at CHR6:86209022 in exon 4 (Jann et al., 2004).

CSN3*J varies from the reference CSN3*B in the substitution of the amino acid Tyr_61_ to Cys_61_ because of the transition of A to G in exon 4 at CHR6:86209011. (Jann et al., 2004).

CSN3*K distinguishes from the reference variant by the amino acid substitution Gln_44_ to Arg_44_ that is caused by an A to G transition at CHR6:86208960 in exon 4 (Jann et al., 2004). CSN3*K and CSN3*I were found at a frequency ≤0.10 in Bunte and Weise Deutsche Edelziege, and Buren goats from Germany (Kupper et al., 2010). In addition, CSN3*K was detected in Taggar and Saanen goats from Sudan (Rahmatalla et al., 2021).

CSN3*L contains the same amino acid substitution Gln_44_ to Arg_44_ that occurs in CSN3*K. The additional substitution of Ser_159_ by Pro_159_ differentiate CSN3*L from the reference. This substitution to Pro_159_ has been detected before in the other protein variants CSN3*C, CSN3*D, CSN3*F, and CSN3*G (Kiplagat et al., 2010; Gautam et al., 2019). CSN3*L had been named before as CSN3*D (Kiplagat et al., 2010). This protein variant was found in Afar goats from Ethiopia only at a frequency of 0.05 (Kiplagat et al., 2010).

CSN3*M (provisionally named CSN3*Y) differs from the reference protein by three non-synonymous mutations. CSN3*M is characterized by a G to A transition at CHR6:86209097 resulting in the amino acid exchange of Asp_90_ to Asn_90_, a T to C transition at CHR6:86209263 resulting in a Val_145_ to Ala_145_ substitution, and a T to C transition at CHR6:86209304 resulting in the amino acid change from Ser_159_ to Pro_159_ (Chessa et al., 2003; Prinzenberg et al., 2005). CSN3*M occurs at a frequency of ≤0.05 in few Italian goats and Red Sokoto goats from Cameroon and Nigeria (Supplementary Table S6) (Chessa et al., 2003; Prinzenberg et al., 2005; Sacchi et al., 2005; Caroli et al., 2007; Gigli et al., 2008; Vacca et al., 2014). CSN3*M′ (previously named CSN3*N) differs from the variant CSN3*M by a synonymous mutation in codon 156 (Kiplagat et al., 2010; Gautam et al., 2019). CSN3*M′ occurs at a frequency of 0.01 in Short Eared goats from Somalia (Kiplagat et al., 2010).

CSN3*N, named before as CSN3*M, carries the two amino acid substitutions Asp_90_ to Asn_90_ and Val_145_ to Ala_145_ (Kiplagat et al., 2010; Gautam et al., 2019).

The CSN3*O protein variant (previously CSN3*Q) carries the substitutions of Val_65_ to Ile_65_, Ile_119_ to Val_119_ and Ser_159_ to Pro_159_ compared to the reference (Kiplagat et al., 2010; Gautam et al., 2019). CSN3*O and CSN3*N found in a few goats from Africa at a frequency of less than 0.05 and 0.15, respectively (Kiplagat et al., 2010; Rahmatalla et al., 2021).

The replacements of Gln_44_ to Arg_44_ and Val_65_ to Ile_65_ lead to the variant CSN3*P (Gautam et al., 2019).

CSN3*Q carries the substitutions Gln_44_ to Arg_44_, Ile_119_ to Val_119_ and Ser_159_ to Pro_159,_ that had been detected and described before in other k-casein variants. Furthermore, CSN3*Q has the amino acid substitution Thr_82_ to Ala_82_ which is the result of an A to G transition at CHR6:86209073 in exon 4 (Gautam et al., 2019). CSN3*Q and CSN3*P were found at a frequency of 0.01 in Blank Bengal Indian goats (Gautam et al., 2019).

CSN3*R distinguishes from the reference in the amino acids Pro_80_ to Leu_80_ and Ile_119_ to Val_119_. The exchanges from Pro_80_ to Leu_80_ and Ile_119_ to Val_119_ result from a transition of C to T and A to G in exon 4 at CHR6:86,209,068 and CHR6:86209184, respectively (Gautam et al., 2019).

CSN3*S is distinguished from the reference by the amino acid substitutions Val_67_ to Ala_67_ and Ile_119_ to Val_119_. The substitution of Val_67_ to Ala_67_ is a result of a T to C transition at CHR6:86209029 in exon 4 (Gautam et al., 2019). The frequency of CSN3*S and CSN3*R is about 0.01 in Siroh goats from India (Gautam et al., 2019).

CSN3*T carries also the Ile_119_ to Val_119_ substitution. In addition, the substitution Ser_37_ to Arg_37_ is present which results from an A to C transversion at CHR6:86208938 in exon 4 (Gautam et al., 2019).

CSN3*U varies from the reference by two amino acid substitutions, Val_78_ to Ala_78_ and Asn_143_ to His_143_. The substitution of Val_78_ to Ala_78_ results from the mutation TT to CA at CHR6:86209062 and CHR6:86209063; the substitution of Asn_143_ to His_143_ occurs due to an A to C transversion at CHR6:86209256 in exon 4 (Gautam et al., 2019).

CSN3*V variant carries the Asn_53_ to Ser_53_ substitution that occurs also in variant CSN3*H, but variant CSN3*V contains also the amino acid change from Ser_159_ to Pro_159_ that can be found also in other k-casein variants (Gautam et al., 2019).

In CSN3*W, the amino acid substitution Asn_53_ to Ser_53_ is found as in CSN3*H and CSN3*V. In addition, the amino acid substitution Thr_138_ to Ala_138_ as a result of an A to G transition at CHR6:86209241 occurs in CSN3*W (Gautam et al., 2019). The protein variants CSN3*H, CSN3*T, CSN3*U, CSN3*V, and CSN3*W are rare. They were found at a frequency of 0.01 in Rajgarh Indian breed (Gautam et al., 2019).

CSN3*X differs from the reference by the amino acid substitutions Ser_33_ to Asn_33_ and Ser_37_ to Thr_37_ which are the result of G to A transitions at CHR6:86208927 and CHR6:86208939, respectively. CSN3*X was identified in Alpine ibex (Rahmatalla et al., 2021).

No information is available for the allele frequency of CSN3*A′, CSN3*B″, CSN3*J, and CSN3*X.

The *CSN3* protein variants are classified into two groups based on the isoelectric focusing point (IEP). CSN3*A, CSN3*B, CSN3*B′, CSN3*B″, CSN3*C, CSN3*C′, CSN3*F, CSN3*G, CSN3*H, CSN3*I, CSN3*J, CSN3*L have an isoelectric focusing point of 5.29 and belong to group A^IEF^. CSN3*D, CSN3*E, CSN3*K, CSN3*M have an isoelectric focusing point of 5.66 and belong to group B^IEF^ (Prinzenberg et al., 2005). The IEF pattern of CSN3*D′ and CSN3*N were not experimentally tested, but estimated using the ExPASy tool (Gasteiger et al., 2003). The D′ variant was classified to group B^IEF^ and the N variant (new name G”) was classified to group A^IEF^ (Gerlando et al., 2015). The k-casein variants in group B^IEF^ were significantly associated with higher milk casein and protein content, which was shown in different goat breeds (Chiatti et al., 2005; Chiatti et al., 2007; Caravaca et al., 2009).

### Casein haplotypes

Due to the strong genetic linkage of the four casein genes within a cluster of 250 kb, the haplotype approach has been a suitable tool instead of evaluating a single gene for accessing the variability within and among populations in diversity studies (Sacchi et al., 2005; Finocchiaro et al., 2008; Gigli et al., 2008; Kupper et al., 2010; Criscione et al., 2019), for breeding strategies (Haplotype Assisted Selection) (Caroli et al., 2006; Hayes et al., 2006), and for prediction of individual milk composition and suitability for milk processing. However, the full definition of the casein haplotypes would require genomic sequence data and is also complicated because of the large number of genetic variations found at the four casein loci. One of the major problems in comparing the different research when considering haplotypes is the different mutations used for deriving casein haplotypes. Furthermore, the number of genotyped animals per population or breeds, and the availability of pedigree and parent information affect the reconstructing and thereby the accuracy of the exact haplotypes carried by each animal.

The evaluation of the haplotype variability in five Italian goat breeds from Northern and Southern Italy identified 18 common haplotypes (Sacchi et al., 2005). However, the study failed to genotype CSN2*C which, therefore, could not be considered it in the haplotype construction. The haplotype CSN1S1*F-CSN1S2*F-CSN3*D occurred in all breeds and was the most common haplotype in the Southern Italian breeds. In the Northern Italian breed, the haplotypes CSN1S1*E-CSN1S2*C-CSN3*D and CSN1S1*F-CSN1S2*C-CSN3*D were most prevalent. A high frequency of the CSN1S1*0-CSN1S2*C-CSN3*A haplotype, containing the null variant CSN1S1*0, was detected in Vallesana Italian goats. Interestingly, the haplotype CSN1S1*F-CSN2*0, which was found in Maltese goats, was associated with a very low casein level of around 2.5 g/L, even if it was linked to a strong variant of *CSN1S2*. Eventually, the “Maltese” haplotype is the result of selection for milk with specific nutritional properties rather than for cheese-making (Sacchi et al., 2005).

In a study of Caroli et al. (2006) in four goat breeds from Northern Italy, 13 haplotypes were identified that occurred at a frequency >0.05 in at least one breed. Based on the evolution model, CSN1S1*B-CSN2*A-CSN1S2*A-CSN3*B was suggested to be the ancestral haplotype (Caroli et al., 2006).

Fifty four haplotypes were detected by using 30 SNPs within the casein genes in the analysis of six Southern Italian breeds and Norwegian dairy goat which highlighted the significant diversity among the breed (Criscione et al., 2019).

The haplotype structure within each casein gene was constructed in Norwegian goats based on 39 SNPs (Hayes et al., 2006). The unique number of haplotypes were 10, 6, 4, and 8 for *CSN1S1*, *CSN2*, *CSN1S2*, and *CSN3*, respectively. Since *CSN1S1* haplotypes significantly affected the protein percentage and fat yield, and *CSN3* haplotypes the protein and fat percentage, the authors suggest the use of a haplotype-assisted selection approach in Norwegian dairy goats, which are used specifically for cheese production (Hayes et al., 2006).

The haplotype structure was based on 22 SNPs and one deletion within the four casein genes in two geographically distant populations, the Sicilian Girgentana breed that likely originated from Afghanistan and the Himalaya area and the Norwegian goat breed (Finocchiaro et al., 2008). The number of haplotypes were 10, 8, 3, and 11 for *CSN1S1*, *CSN2*, *CSN1S2*, and *CSN3*, respectively. Although considerably geographically distant and phenotypically divergent, casein haplotypes were identical between both breeds.

In five Italian Sicilian goat breeds, 27 haplotypes were observed at a frequency >0.03. The most frequent haplotype in Derivata di Siria and Maltese was CSN1S1*F-CSN2*C-CSN1S2*F-CSN3*B. This haplotype is associated with low milk casein content, and therefore superior for the production of hypoallergenic goat milk (Gigli et al., 2008). In Argentata dell’Etna, Girgentana and Messinese the most frequent haplotype was CSN1S1*A-CSN2*C-CSN1S2*A-CSN3*B which is also most favorable for cheese production (Gigli et al., 2008).

In Bunte Deutsche Edelziege and Weiße Deutsche Edelziege dairy goats from Germany, 30 haplotypes with a frequency higher than 0.01 were detected, only four haplotypes were found in Buren meat goats (Kupper et al., 2010). The ancestral haplotype CSN1S1*B-CSN2*A-CSN1S2*A-CSN3*B was evident in all three breeds with the highest frequency in Buren goats (Kupper et al., 2010). The CSN1S1*E-CSN2*A-CSN1S2*A-CSN3*B haplotype was most frequent in both dairy breeds followed by CSN1S1*F-CSN2*C-CSN1S2*F-CSN3*B in Bunte Deutsche Edelziege and CSN1S1*F-CSN2*C1-CSN1S2*F-CSN3*B in Weiße Deutsche Edelziege, which might be attributed to the selection of milk quantity. The deep divergence in the casein gene haplotypes in dairy goat breeds was attributed to different selection aims among breeds.

### Application of casein variants for genetic selection programs

The selection for favorable alleles of major genes within a breed is cost-effective if it can be implemented in an existing selection system. Major genes affecting milk protein content, particularly caseins, are well known. The prevalence of specific casein variants in different goat breeds is probably the result of breeding goals but could be also due to random fixation. Currently, there are several kits on the market for testing milk protein variants in goats (Simões and Gutiérrez, 2018). Genotyping *CSN1S1* is of particular interest for breeders and the milk industry since genetic variation in the *CSN1S1* gene is one of the primary factors affecting milk protein content and technological properties, such as cheese yield and cheese curd formation. Selection schemes in Alpine and Saanen goats currently use *CSN1S1* variants for the pre-selection of young males undergoing the progeny test (Manfredi et al., 2000).

It is also conceivable to select goats carrying casein gene variants with anti-allergenic properties to produce healthy milk as functional food for human nutrition (Robinson, 2001).

### Impact of casein variants in goat milk on human health and nutrition

Goat milk proteins possess unique biologically active components that affect the milk properties and have an impact on numerous aspects of human nutrition. Goat milk contains less lactose and higher alkalinity compared to cow milk (Robinson, 2011). Goat milk is highly digestible and has been approved repeatedly as an alternative milk source for people, who suffer from lactose intolerance and cow milk protein allergies (Park and Haenlein, 2017). Therefore, goat milk has a therapeutic value in human medicine and nutrition (Park, 2009).

Many people who are allergic and have gastrointestinal disorders to cow milk can tolerate goat milk because of considerable variation in goat milk protein sequences (Park and Haenlein, 2017). Numerous cow milk proteins have been identified as allergens (Wal, 2002). Alpha-S1-casein, α-S2-casein, and beta-lactoglobulin are among the most crucial milk protein allergens (Wal, 1998; Crittenden and Bennett, 2005). Beta-lactoglobulin does not occur in human milk (O'Mahony et al., 2014). For example, α-S1-casein from cow milk forms hard curds in the stomach which can cause digestion problems. Therefore, the advantage of goat milk is the low concentration or even null content of α-S1-casein and other allergic casein protein variants (Bernacka, 2011; Yangilar, 2013).

For instance, the association of the casein protein variants with the null or low content of those proteins might affect the production of goat milk with hypoallergenic characteristics and selecting milk with null or reduced content of the particular protein. In this concern, some variants in goat α-S1-casein, β-casein, and α-S2-casein in different goat breeds are associated with null or reduced expression of the encoded protein (Persuy et al., 1999; Rando et al., 2000; Ramunno et al., 2001a; Ramunno et al., 2001b; Ramunno et al., 2005; Caroli et al., 2006).

Through the digestion of goat milk proteins and milk products, several interesting peptides have been identified with bioactive properties. The beneficial health effects of these peptides include antihypertensive, anti-diabetic, antithrombotic, immunomodulatory, antibacterial, antifungal, antiviral, antioxidant, binding and transporting metals, preventing amnesia, and causing smooth muscle contractions properties (Ebringer et al., 2008; Atanasova and Ivanova, 2010; Zhang et al., 2015; Gong et al., 2020).

## Conclusion

In this review, we provide a comprehensive overview on the genetic variation of the four casein proteins in different goat breeds. We provide a translation of genetic DNA variants to the corresponding amino acid changes, as well as the protein variants. In addition, we also updated the names of different protein variants according to the information we found in a literature search on reported protein and genetic variants in casein to summarize more than 30 years of research. The effect of different casein gene variants on the casein content, milk composition, and cheese-making properties is discussed. The wide variation occurring in the milk caseins has impact on the nutritional value for humans and on technological properties in milk processing. The different characteristics of goat milk likely affected breeding goals and adaptation, which led to high frequency of some casein variants in various goat breeds and country. The casein genomic and proteomic variations as well as the casein haplotypes approach might be useful in selection of the animals according to the specific breeding goals.

## Supplementary material

Supplementary Table S1 Nucleotide and amino acid variants within the gene and mature protein of the *CSN1S1*.

Supplementary Table S2 Frequency of *CSN1S1* variants in different goat breeds and countries.

Supplementary Table S3 Frequency of *CSN2* variants in different goat breeds and countries.

Supplementary Table S4 Frequency of *CSN1S2* variants in different goat breeds and countries.

Supplementary Table S5 Nucleotide and amino acid variants within the gene and mature protein of the *CSN3*.

Supplementary Table S6 Frequency of *CSN3* variants in different goat breeds and countries.

## References

[B1] Albenzio M. Santillo A. D'angelo F. Sevi A. (2009). Focusing on casein gene cluster and protein profile in Garganica goat milk. J. Dairy Res. 76, 83–89. 10.1017/S0022029908003853 19121241

[B2] Alexander L. J. Stewart A. F. Mackinlay A. G. Kapelinskaya T. V. Tkach T. M. Gorodetsky S. I. (1988). Isolation and characterization of the bovine kappa-casein gene. Eur. J. Biochem. 178, 395–401. 10.1111/j.1432-1033.1988.tb14463.x 3208764

[B3] Ambrosoli R. Di Stasio L. Mazzocco P. (1988). Content of alpha S1-casein and coagulation properties in goat milk. J. Dairy Sci. 71, 24–28. 10.3168/jds.S0022-0302(88)79520-X 3372804

[B4] Angiolillo A. Yahyaoui M. H. Sanchez A. Pilla F. Folch J. M. Sanchez A. (2002). Short communication: Characterization of a new genetic variant in the caprine kappa-casein gene. J. Dairy Sci. 85, 2679–2680. 10.3168/jds.s0022-0302(02)74353-1 12416822

[B5] Aschaffenburg R. Drewry J. (1955). Occurrence of different beta-lactoglobulins in cow's milk. Nature 176, 218–219. 10.1038/176218b0 13244664

[B6] Assan N. (2014). Factors affecting yield and milk composition in dairy animals. Sunnyvale, CA, USA: Lap Lambert Academic Publishing GmbH KG.

[B7] Atanasova J. Ivanova I. (2010). Antibacterial peptides from goat and sheep milk proteins. Biotechnol. Biotechnol. Equip. 24, 1799–1803. 10.2478/V10133-010-0049-8

[B8] Baltrėnaitė L. Kerzienė S. Morkūnienė K. Miceikienė I. (2009). Genetic diversity in milk proteins among goats bred in Lithuania. Acta Univ. Latv. 753, 117–124.

[B9] Barth C. A. Schlimme E. (2012). Milk proteins: Nutritional, clinical, functional and technological aspects. Heidelberg, Germany: Steinkopff Heidelberg.

[B10] Bernacka H. (2011). Health-promoting properties of goat milk. Med. Wet. 67.

[B11] Bevilacqua C. Ferranti P. Garro G. Veltri C. Lagonigro R. Leroux C. (2002). Interallelic recombination is probably responsible for the occurrence of a new alpha(s1)-casein variant found in the goat species. Eur. J. Biochem. 269, 1293–1303. 10.1046/j.1432-1033.2002.02777.x 11856364

[B12] Bhat M. Y. Dar T. A. Laishram Rajendrakumar Singh S. K. (2016). “Casein proteins: Structural and functional aspects,” in Milk proteins. Editor Gigli I. (London, UK: IntechOpen), 296.

[B13] Bickhart D. M. Rosen B. D. Koren S. Sayre B. L. Hastie A. R. Chan S. (2017). Single-molecule sequencing and chromatin conformation capture enable de novo reference assembly of the domestic goat genome. Nat. Genet. 49, 643–650. 10.1038/ng.3802 28263316 PMC5909822

[B14] Birgisdottir B. E. Hill J. P. Harris D. P. Thorsdottir I. (2002). Variation in consumption of cow milk proteins and lower incidence of Type 1 diabetes in Iceland vs the other 4 Nordic countries. Diabetes Nutr. Metab. 15, 240–245. 12416661

[B15] Boulanger A. Grosclaude F. Mahé M. F. (1984). Polymorphisme des caséines α(s1 )et α(s2 )de la chèvre (*Capra hircus*). Genet. Sel. Evol. 16, 157–176. 10.1186/1297-9686-16-2-157 PMC2714354 22879157

[B16] Bouniol C. Brignon G. Mahe M. F. Printz C. (1994). Biochemical and genetic analysis of variant C of caprine alpha s2-casein (*Capra hircus*). Anim. Genet. 25, 173–177. 10.1111/j.1365-2052.1994.tb00106.x 7943951

[B17] Bouniol C. Brignon G. Mahe M. F. Printz C. (1993). Characterization of goat αs2 casein A and B: Further evidence of the phosphorylation code of caseins. Protein Sequence Data Analysis 5, 213–218.

[B18] Bouniol C. (1993). Sequence of the goat αs2-casein-encoding cDNA. Gene 125, 235–236. 10.1016/0378-1119(93)90336-2 8462880

[B19] Brignon G. Mahe M. F. Grosclaude F. Ribadeau-Dumas B. (1989). Sequence of caprine alpha s1-casein and characterization of those of its genetic variants which are synthesized at a high level, alpha s1-CnA, B and C. Protein Seq. Data Anal. 2, 181–188. 2755948

[B20] Brignon G. Mahe M. F. Ribadeau-Dumas B. Mercier J. C. Grosclaude F. (1990). Two of the three genetic variants of goat alpha s1-casein which are synthesized at a reduced level have an internal deletion possibly due to altered RNA splicing. Eur. J. Biochem. 193, 237–241. 10.1111/j.1432-1033.1990.tb19328.x 2226443

[B21] Caravaca F. Amills M. Jordana J. Angiolillo A. Agüera P. Aranda C. (2008). Effect of alphas1-casein ( CSN1S1) genotype on milk CSN1S1 content in Malagueña and Murciano-Granadina goats. J. Dairy Res. 75, 481–484. 10.1017/S0022029908003609 19032797

[B22] Caravaca F. Ares J. L. Carrizosa J. Urrutia B. Baena F. Jordana J. (2011). Effects of alpha s1-casein (CSN1S1) and kappa-casein (CSN3) genotypes on milk coagulation properties in Murciano-Granadina goats. J. Dairy Res. 78, 32–37. 10.1017/S002202991000083X 21214964

[B23] Caravaca F. Carrizosa J. Urrutia B. Baena F. Jordana J. Amills M. (2009). Short communication: Effect of alphaS1-casein (CSN1S1) and kappa-casein (CSN3) genotypes on milk composition in Murciano-Granadina goats. J. Dairy Sci. 92, 2960–2964. 10.3168/jds.2008-1510 19448028

[B24] Caroli A. Chiatti F. Chessa S. Rignanese D. Bolla P. Pagnacco G. (2006). Focusing on the goat casein complex. J. Dairy Sci. 89, 3178–3187. 10.3168/jds.S0022-0302(06)72592-9 16840635

[B25] Caroli A. Chiatti F. Chessa S. Rignanese D. Ibeagha-Awemu E. M. Erhardt G. (2007). Characterization of the casein gene complex in West African goats and description of a new alpha(s1)-casein polymorphism. J. Dairy Sci. 90, 2989–2996. 10.3168/jds.2006-674 17517740

[B26] Caroli A. Jann O. Budelli E. Bolla P. Jager S. Erhardt G. (2001). Genetic polymorphism of goat kappa-casein (CSN3) in different breeds and characterization at DNA level. Anim. Genet. 32, 226–230. 10.1046/j.1365-2052.2001.00765.x 11531704

[B27] Caroli A. M. Chessa S. Erhardt G. J. (2009). Invited review: Milk protein polymorphisms in cattle: Effect on animal breeding and human nutrition. J. Dairy Sci. 92, 5335–5352. 10.3168/jds.2009-2461 19841193

[B28] Catota-Gómez L. D. Parra-Bracamonte G. M. Cienfuegos-Rivas E. G. Hernández-Meléndez J. Sifuentes-Rincón A. M. Martínez-González J. C. (2017). Frequency and association of polymorphisms in CSN3 gene with milk yield and composition in Saanen goats. Ecosist. Recur. Agropec. 4, 411–417. 10.19136/era.a4n12.1165

[B29] Cebo C. Lopez C. Henry C. Beauvallet C. Ménard O. Bevilacqua C. (2012). Goat αs1-casein genotype affects milk fat globule physicochemical properties and the composition of the milk fat globule membrane. J. Dairy Sci. 95, 6215–6229. 10.3168/jds.2011-5233 22921619

[B30] Chanat E. Martin P. Ollivier-Bousquet M. (1999). Alpha(S1)-casein is required for the efficient transport of beta- and kappa-casein from the endoplasmic reticulum to the Golgi apparatus of mammary epithelial cells. J. Cell. Sci. 112, 3399–3412. 10.1242/jcs.112.19.3399 10504344

[B31] Chessa S. Bolla P. (2003). Il polimorfismo della caseine caprine: Uno straordinario “puzzle” genetico dalle svariate potenzialità applicative. Sci. Tecn. Latt.-Cas. 54, 343–358.

[B32] Chessa S. Budelli E. Chiatti F. Cito A. M. Bolla P. Caroli A. (2005). Short communication: Predominance of beta-casein (CSN2) C allele in goat breeds reared in Italy. J. Dairy Sci. 88, 1878–1881. 10.3168/jds.S0022-0302(05)72863-0 15829682

[B33] Chessa S. Budelli E. Gutscher K. Caroli A. Erhardt G. (2003). Short communication: Simultaneous identification of five kappa-casein (CSN3) alleles in domestic goat by polymerase chain reaction-single strand conformation polymorphism. J. Dairy Sci. 86, 3726–3729. 10.3168/jds.S0022-0302(03)73978-2 14672203

[B34] Chessa S. Chiatti F. Rignanese D. Ibeagha-Awemu E. M. Özbeyaz C. Hassan Y. A. (2007). The casein genes in goat breeds from different continents: Analysis by polymerase chain reaction – single strand conformation polymorphism (PCR-SSCP). Italian J. Animal Sci. 6, 73–75. 10.4081/ijas.2007.1s.73

[B35] Chessa S. Rignanese D. Kupper J. Pagnacco G. Erhardt G. Caroli A. (2008). Short communication: The beta-casein (CSN2) silent allele C1 is highly spread in goat breeds. J. Dairy Sci. 91, 4433–4436. 10.3168/jds.2008-1228 18946150

[B36] Chianese L. Caira S. Garro G. Quarto M. Mauriello R. Addeo F. (2007). “Occurrence of genetic polymorphism at goat β-CN locus,” in 5th Int. Symp, Challenge to Sheep and Goats Milk Sectors, Sardinia, Italy.

[B37] Chianese L. Ferranti P. Garro G. Mauriello R. Addeo F. (1997). “Occurrence of three novel alpha s1-casein variants in goat milk,” in International Dairy Federation, Brussels, Belgium.

[B38] Chianese L. Garro G. Ferranti P. Speranza L. M. Pilla F. Bevilacqua C. (1998). “Occurence of new variant ofgoat αs1-and αs2-casein in abreed of Southern Italy,” in Proceeding of 25th International Dairy Congress, 82nd annual sessions, Arahus, Denmark.

[B39] Chiatti F. Caroli A. Chessa S. Bolla P. Pagnacco G. (2005). “Relationships between goat κ-casein (CSN3) polymorphism and milk composition,” in The role of biotechnology. Villa Gualino, Turin, Italy: AGRIS.

[B40] Chiatti F. Chessa S. Bolla P. Cigalino G. Caroli A. Pagnacco G. (2007). Effect of kappa-casein polymorphism on milk composition in the Orobica goat. J. Dairy Sci. 90, 1962–1966. 10.3168/jds.2006-508 17369237

[B41] Cieslinska A. Kostyra E. Kostyra H. Olenski K. Fiedorowicz E. Kaminski S. (2012). Milk from cows of different beta-casein genotypes as a source of beta-casomorphin-7. Int. J. Food Sci. Nutr. 63, 426–430. 10.3109/09637486.2011.634785 22080615

[B42] Clark S. Mora Garcia M. B. (2017). A 100-Year Review: Advances in goat milk research. J. Dairy Sci. 100, 10026–10044. 10.3168/jds.2017-13287 29153153

[B43] Clark S. Sherbon J. W. (2000). Alpha s1-casein, milk composition and coagulation properties of goat milk. Small Ruminant Res. 38, 123–134. 10.1016/S0921-4488(00)00154-1

[B44] Coll A. Folch J. M. Sanchez A. Sanchez A. (1993). Nucleotide sequence of the goat kappa-casein cDNA. J. Anim. Sci. 71, 2833. 10.2527/1993.71102833x 8226388

[B45] Cosenza G. Gallo D. Illario R. Di Gregorio P. Senese C. Ferrara L. (2003). A Mval PCR-RFLP detecting a silent allele at the goat alpha-lactalbumin locus. J. Dairy Res. 70, 355–357. 10.1017/s0022029903006265 12916831

[B46] Cosenza G. Iannaccone M. Pico B. A. Ramunno L. Capparelli R. (2016). The SNP g.1311T>C associated with the absence of β-casein in goat milk influences CSN2 promoter activity. Anim. Genet. 47, 615–617. 10.1111/age.12443 27392512

[B47] Cosenza G. Pauciullo A. Colimoro L. Mancusi A. Rando A. Di Berardino D. (2007). An SNP in the goat CSN2 promoter region is associated with the absence of β-casein in milk. Anim. Genet. 38, 655–658. 10.1111/j.1365-2052.2007.01649.x 17931404

[B48] Cosenza G. Pauciullo A. Gallo D. Berardino D. D. Ramunno L. (2005). A Ssp I PCR-RFLP detecting a silent allele at the goat CSN2 locus. J. Dairy Res. 72, 456–459. 10.1017/S0022029905001342 16223461

[B49] Cosenza G. Pauciullo A. Gallo D. Colimoro L. D’avino A. Mancusi A. (2008). Genotyping at the CSN1S1 locus by PCR-RFLP and AS-PCR in a Neapolitan goat population. Small Ruminant Res. 74, 84–90. 10.1016/j.smallrumres.2007.03.010

[B50] Criscione A. Tumino S. Avondo M. Marletta D. Bordonaro S. (2019). Casein haplotype diversity in seven dairy goat breeds. Arch. Anim. Breed. 62, 447–454. 10.5194/aab-62-447-2019 31807656 PMC6853139

[B51] Crittenden R. G. Bennett L. E. (2005). Cow's milk allergy: A complex disorder. J. Am. Coll. Nutr. 24, 582S–591S. 10.1080/07315724.2005.10719507 16373958

[B52] Cunsolo V. Muccilli V. Saletti R. Marletta D. Foti S. (2006). Detection and characterization by high-performance liquid chromatography and mass spectrometry of two truncated goat alphas2-caseins. Rapid Commun. Mass Spectrom. 20, 1061–1070. 10.1002/rcm.2415 16617471

[B53] Dall’olio S. Davoli R. Russ O. V. (1989). Una nuova variante di caseina caprina. Sci. Tec. Latt. Casearia 40, 24–28.

[B54] De Noni I. Cattaneo S. (2010). Occurrence of β-casomorphins 5 and 7 in commercial dairy products and in their digests following *in vitro* simulated gastro-intestinal digestion. Food Chem. 119, 560–566. 10.1016/j.foodchem.2009.06.058

[B55] Delacroix Buchet A. Josas J. E. Laitieres R. Degas C. Lamberet G. Vassal L. (1996). Effect of AA and FF caprine αs1-casein variants on cheesemaking. Lait 76, 217–241. 10.1051/lait:1996319

[B56] Devold T. G. Nordb R. Langsrud T. Svenning C. Brovold M. J. Sørensen E. S. (2011). Extreme frequencies of the αs1-casein “null” variant in milk from Norwegian dairy goats— Implications for milk composition, micellar size and renneting properties. Dairy Sci. Technol. 91, 39–51. 10.1051/dst/2010033

[B57] Ebringer L. Ferencik M. Krajcovic J. (2008). Beneficial health effects of milk and fermented dairy products--review. Folia Microbiol. (Praha). 53, 378–394. 10.1007/s12223-008-0059-1 19085072

[B58] El-Shazly S. A. Ahmed M. M. Amer S. M. (2017). Genetic polymorphism in some milk protein genes and its impact on milk composition of Saudi Arabian goat breeds reared in Taif region. Romanian Biotechnol. Lett. 22.

[B59] Elagamy E. I. (2016). “Milk protein allergy,” in Reference module in food science.

[B60] Erhardt G. Jäger S. Budelli E. Caroli A. (2002). Genetic polymorphism of goat αS2-casein (CSN1S2) and evidence for a further allele. Milchwissenschaft 57, 137–140.

[B61] Farrell H. M. Jimenez-Flores R. Bleck G. T. Brown E. M. Butler J. E. Creamer L. K. (2004). Nomenclature of the proteins of cows’ milk—sixth revision. J. Dairy Sci. 87, 1641–1674. 10.3168/jds.S0022-0302(04)73319-6 15453478

[B62] Ferretti L. Leone P. Sgaramella V. (1990). Long range restriction analysis of the bovine casein genes. Nucleic Acids Res. 18, 6829–6833. 10.1093/nar/18.23.6829 2263448 PMC332738

[B63] Finocchiaro R. Hayes B. J. Siwek M. Spelman R. J. Van Kaam J. B. Adnoy T. (2008). Comparison of casein haplotypes between two geographically distant European dairy goat breeds. J. Anim. Breed. Genet. 125, 68–72. 10.1111/j.1439-0388.2007.00687.x 18254829

[B64] Fox P. F. Brodkorb A. (2008). The casein micelle: Historical aspects, current concepts and significance. Int. Dairy J. 18, 677–684. 10.1016/j.idairyj.2008.03.002

[B65] Fox P. F. Mcsweeney P. L. H. (1998). Dairy chemistry and biochemistry. Berlin, Germany: Springer.

[B66] Galliano F. Saletti R. Cunsolo V. Foti S. Marletta D. Bordonaro S. (2004). Identification and characterization of a new beta-casein variant in goat milk by high-performance liquid chromatography with electrospray ionization mass spectrometry and matrix-assisted laser desorption/ionization mass spectrometry. Rapid Commun. Mass Spectrom. 18, 1972–1982. 10.1002/rcm.1575 15329864

[B67] Gasteiger E. Gattiker A. Hoogland C. Ivanyi I. Appel R. D. Bairoch A. (2003). ExPASy: The proteomics server for in-depth protein knowledge and analysis. Nucleic Acids Res. 31, 3784–3788. 10.1093/nar/gkg563 12824418 PMC168970

[B68] Gautam D. Vats A. Verma M. Rout P. K. Meena A. S. Ali M. (2019). Genetic variation in CSN3 exon 4 region of Indian goats and a new nomenclature of CSN3 variants. Anim. Genet. 50, 191–192. 10.1111/age.12767 30724371

[B69] Gerlando R. D. Tortorici L. Sardina M. T. Monteleone G. Mastrangelo S. Portolano B. (2015). Molecular characterisation of κ–casein gene in Girgentana dairy goat breed and identification oftwo new alleles. Italian J. Animal Sci. 14, 3464. 10.4081/ijas.2015.3464

[B70] Ghosh D. Das S. Bagchi D. Smarta R. B. (2016). Innovation in healthy and functional foods. Boca Raton, FL, USA: CRC Press.

[B71] Giambra I. J. Erhardt G. E. (2012). Milk proteins, milk protein variants and the importance for the sheep breeding - a review. Züchtungskunde. 84, 52–73.

[B72] Gigli I. Maizon D. O. Riggio V. Sardina M. T. Portolano B. (2008). Short communication: Casein haplotype variability in Sicilian dairy goat breeds. J. Dairy Sci. 91, 3687–3692. 10.3168/jds.2008-1067 18765627

[B73] Gigli I. (2016). Milk proteins: From structure to biological properties and health aspects. IntechOpen.

[B74] Glantz M. Devold T. G. Vegarud G. E. Lindmark Mansson H. Stalhammar H. Paulsson M. (2010). Importance of casein micelle size and milk composition for milk gelation. J. Dairy Sci. 93, 1444–1451. 10.3168/jds.2009-2856 20338421

[B75] Gong H. Gao J. Wang Y. Luo Q. W. Guo K. R. Ren F. Z. (2020). Identification of novel peptides from goat milk casein that ameliorate high-glucose-induced insulin resistance in HepG2 cells. J. Dairy Sci. 103, 4907–4918. 10.3168/jds.2019-17513 32253041

[B76] Grosclaude F. Mahe M. F. Brignon G. Di Stasio L. Jeunet R. (1987). A Mendelian polymorphism underlying quantitative variations of goat α(s1)-casein. Genet. Sel. Evol. 19, 399–412. 10.1186/1297-9686-19-4-399 PMC2713294 22879295

[B77] Grosclaude F. Martin P. (1997). “Casein polymorphisms in the goat,” in International dairy federation (Palmerston North, New Zealand: IDF Seminar `Milk Protein Polymorphism II).

[B78] Grosclaude F. Ricordeau G. Martin P. Remeuf F. Vassal L. Bouillon J. (1994). Du gène au fromage : Le polymorphisme de la caséine alphas1 caprine, ses effets, son évolution. INRA. Prod. Anim. 7, 3–19. 10.20870/productions-animales.1994.7.1.4153

[B79] Guinee T. P. O'brien B. (2010). “The quality of milk for cheese manufacture,” in Technology of cheesemaking. Editors Law B. A. Tamime A. Y., 1–67.

[B80] Guo M. (2009). “Chapter 9 - human milk and infant formula,” in Functional foods. Editor Guo M. (Sawston, UK: Woodhead Publishing), 299–337.

[B81] Haratifar S. Guri A. (2017). “5-Nanocapsule formation by caseins,” in Nanoencapsulation technologies for the food and nutraceutical industries. Editor Jafari S. (Oxford: Academic Press), 140–164.

[B82] Hayes B. Hagesaether N. Adnoy T. Pellerud G. Berg P. R. Lien S. (2006). Effects on production traits of haplotypes among casein genes in Norwegian goats and evidence for a site of preferential recombination. Genetics 174, 455–464. 10.1534/genetics.106.058966 16849606 PMC1569804

[B83] Hayes H. C. Petit E. J. (1993). Mapping of the beta-lactoglobulin gene and of an immunoglobulin M heavy chain-like sequence to homoeologous cattle, sheep, and goat chromosomes. Mamm. Genome 4, 207–210. 10.1007/BF00417564 8499654

[B84] Ho S. Woodford K. Kukuljan S. Pal S. (2014). Comparative effects of A1 versus A2 beta-casein on gastrointestinal measures: A blinded randomised cross-over pilot study. Eur. J. Clin. Nutr. 68, 994–1000. 10.1038/ejcn.2014.127 24986816

[B85] Hui Y. H. Sherkat F. (2005). Handbook of food science, technology, and engineering - 4 Volume Set. Boca Raton, FL, USA: CRC Press.

[B86] Ingham B. Smialowska A. Kirby N. M. Wang C. Carr A. J. (2018). A structural comparison of casein micelles in cow, goat and sheep milk using X-ray scattering. Soft Matter 14, 3336–3343. 10.1039/c8sm00458g 29658047

[B87] Jann O. C. Prinzenberg E. M. Luikart G. Caroli A. Erhardt G. (2004). High polymorphism in the kappa-casein (CSN3) gene from wild and domestic caprine species revealed by DNA sequencing. J. Dairy Res. 71, 188–195. 10.1017/s0022029904000093 15190947

[B88] Jansàpérez M. Leroux C. Bonastre A. S. Martin P. (1994). Occurrence of a LINE sequence in the 3' UTR of the goat alpha s1-casein E-encoding allele associated with reduced protein synthesis level. Gene 147, 179–187. 10.1016/0378-1119(94)90063-9 7926797

[B89] Jianqin S. Leiming X. Lu X. Yelland G. W. Ni J. Clarke A. J. (2016). Effects of milk containing only A2 beta casein versus milk containing both A1 and A2 beta casein proteins on gastrointestinal physiology, symptoms of discomfort, and cognitive behavior of people with self-reported intolerance to traditional cows' milk. Nutr. J. 15, 35. 10.1186/s12937-016-0147-z 27039383 PMC4818854

[B90] Johansson I. Lif Holgerson P. (2011). Milk and oral health. Nestle Nutr. Workshop Ser. Pediatr. Program. 67, 55–66. 10.1159/000325575 21335990

[B91] Johansson M. Högberg M. Andrén A. (2015). Relation between αs1-casein content and coagulation properties of milk from Swedish dairy goats. Open Food Sci. J. 9, 1–4. 10.2174/1874256401509010001

[B92] Jordana J. Amills M. Diaz E. Angulo C. Serradilla J. M. Sanchez A. (1996). Gene frequencies of caprine αs1-casein polymorphism in Spanish goat breeds. Small Ruminant Res. 20, 215–221. 10.1016/0921-4488(95)00813-6

[B93] Kiplagat S. Agaba M. Kosgey I. Okeyo M. Indetie D. Hanotte O. (2010). Genetic polymorphism of kappa-casein gene in indigenous Eastern Africa goat populations. Int. J. Genet. Mol. Biol. 2, 001–005.

[B94] Kolb A. F. Huber R. C. Lillico S. G. Carlisle A. Robinson C. J. Neil C. (2011). Milk lacking alpha-casein leads to permanent reduction in body size in mice. PLoS One 6, e21775. 10.1371/journal.pone.0021775 21789179 PMC3138747

[B95] Kumar A. Rout P. K. Mandal A. Roy R. (2009). Kappa-casein gene polymorphism in Indian goats. ndian J. Biotechnol. 8, 214–221.

[B96] Kumar A. Rout P. K. Mandal A. Roy R. MandAl A. (2007). Identification of the CSN1S1 allele in Indian goats by the PCR-RFLP method. Animal 1, 1099–1104. 10.1017/S1751731107000444 22444854

[B97] Kupper J. Chessa S. Rignanese D. Caroli A. Erhardt G. (2010). Divergence at the casein haplotypes in dairy and meat goat breeds. J. Dairy Res. 77, 56–62. 10.1017/S0022029909990343 19889251

[B98] Kusza S. Ilie D. E. Sauer M. Nagy K. Patras I. Gavojdian D. (2016). Genetic polymorphism of CSN2 gene in Banat white and carpatina goats. Acta Biochim. Pol. 63, 577–580. 10.18388/abp.2016_1266 27382646

[B99] Kusza S. Veress G. Kukovics S. J´Avor A. A. Sanchez A. Angiolillo A. (2007). Genetic polymorphism of s1- and s2-caseins in Hungarian milking goats. Small Ruminant Res. 68, 329–332. 10.1016/j.smallrumres.2005.11.014

[B100] Lagonigro R. Pietrola E. D'andrea M. Veltri C. Pilla F. (2001). Molecular genetic characterization of the goat s2-casein E allele. Anim. Genet. 32, 391–393. 10.1046/j.1365-2052.2001.0781c.x 11736814

[B101] Lamothe S. Robitaille G. St-Gelais D. Britten M. (2007). Short communication: Extraction of beta-casein from goat milk. J. Dairy Sci. 90, 5380–5382. 10.3168/jds.2007-0488 18024728

[B102] Lan X. Chen H. Zhang R. Tian Y. Zhang Y. Fang X. (2005). Association of polymorphisms of CSN1S2 gene with average milk yield and body sizes indexes in Xinong Saanen dairy goat. Acta Veterinaria Zootechnica Sinica 36, 318–322.

[B103] Le Parc A. Leonil J. Chanat E. (2010). AlphaS1-casein, which is essential for efficient ER-to-Golgi casein transport, is also present in a tightly membrane-associated form. BMC Cell. Biol. 11, 65. 10.1186/1471-2121-11-65 20704729 PMC2928771

[B104] Leroux C. Martin P. Mahe M. F. Leveziel H. Mercier J. C. (1990). Restriction fragment length polymorphism identification of goat alpha s1-casein alleles: A potential tool in selection of individuals carrying alleles associated with a high level protein synthesis. Anim. Genet. 21, 341–351. 10.1111/j.1365-2052.1990.tb01979.x 1982486

[B105] Leroux C. Mazure N. Martin P. (1992). Mutations away from splice site recognition sequences might cis-modulate alternative splicing of goat alpha s1-casein transcripts. Structural organization of the relevant gene. J. Biol. Chem. 267, 6147–6157. 10.1016/s0021-9258(18)42674-9 1372900

[B106] López-Expósito I. Amigo L. Recio I. (2008). Identification of the initial binding sites of alphas2-casein f(183-207) and effect on bacterial membranes and cell morphology. Biochim. Biophys. Acta 1778, 2444–2449. 10.1016/j.bbamem.2008.06.018 18655768

[B107] Maga E. A. Daftari P. Kultz D. Penedo M. C. (2009). Prevalence of alphas1-casein genotypes in American dairy goats. J. Anim. Sci. 87, 3464–3469. 10.2527/jas.2009-1854 19648483

[B108] Mahé M. F. Grosclaude F. (1993). Polymorphism of β-casein in the Creole goat of Guadeloupe: Evidence for a null allele. Genet. Sel. Evol. 25, 403. 10.1186/1297-9686-25-4-403

[B109] Mahé M. F. Grosclaude F. (1989). αS1-CnD, another allele associated with a decreased synthesis rate at the caprine αS1-casein locus. Genet. Sel. Evol. 21, 127. 10.1186/1297-9686-21-2-127

[B110] Malkoski M. Dashper S. G. O'brien-Simpson N. M. Talbo G. H. Macris M. Cross K. J. (2001). Kappacin, a novel antibacterial peptide from bovine milk. Antimicrob. Agents Chemother. 45, 2309–2315. 10.1128/AAC.45.8.2309-2315.2001 11451690 PMC90647

[B111] Manfredi E. Serradilla J. M. Leroux C. Martin P. Sánchez A. (2000). “Genetics for milk production,” in Proceedings of the 7th International Conference on Goats (Poitiers, France: INRA, Paris).

[B112] Marković B. Marković M. Trivunović S. Mirecki S. Antunović Z. Veljić M. (2018). Effects of the alpha s1-casein genotype on milk yield and milk composition of Balkan goat in Montenegro. AgricultForest. 64, 5–14. 10.17707/agricultforest.64.3.01

[B113] Marletta D. Bordonaro S. Guastella A. M. Criscione A. D’urso G. (2005). Genetic polymorphism of the calcium sensitive caseins in Sicilian Girgentana and Argentata dell’Etna goat breeds. Small Ruminant Res. 57, 133–139. 10.1016/j.smallrumres.2004.06.019

[B114] Marletta D. Bordonaro S. Guastella A. M. D'urso G. (2004). Genetic polymorphism at CSN1S2 locus in two endangered Sicilian goat breeds. J. Anim. Breed. Genet. 121, 52–56. 10.1046/j.0931-2668.2003.00413.x

[B115] Marletta D. Criscione A. Ordonaro S. B. Guastella A. M. D’urso G. (2007). Review: Casein polymorphism in goat’s milk. Lait 87, 491–504. 10.1051/lait:2007034

[B116] Martin P. Bianchi L. Cebo C. Miranda G. Mcsweeney P. Fox P. (2013). “Genetic polymorphism of milk proteins,” in Advanced dairy chemistry (Boston: Springer).

[B117] Martin P. Leroux C. (1994). “Characterization of a further α_S1_-casein variant generated by exon skipping,” in 24th International Society of Animal Genetics Conference, Prague.

[B118] Martin P. Ollivier-Bousquet M. Grosclaude F. (1999). Genetic polymorphism of caseins: A tool to investigate casein micelle organization. Int. Dairy J. 9, 163–171. 10.1016/S0958-6946(99)00055-2

[B119] Martin P. Szymanowska M. Zwierzchowski L. Leroux C. (2002). The impact of genetic polymorphisms on the protein composition of ruminant milks. Reprod. Nutr. Dev. 42, 433–459. 10.1051/rnd:2002036 12537255

[B120] Mcmahon D. J. Brown R. J. (1984). Composition, structure, and integrity of casein micelles: A review. J. Dairy Sci. 67, 499–512. 10.3168/jds.S0022-0302(84)81332-6

[B121] Mercier J. C. Chobert J. M. Addeo F. (1976). Comparative study of the amino acid sequences of the caseinomacropeptides from seven species. FEBS Lett. 72, 208–214. 10.1016/0014-5793(76)80972-6 16386026

[B122] Mestawet T. A. Girma A. Ådnøy T. Devold T. G. Narvhus J. A. Vegarud G. E. (2014). New insights in goat breeds of Ethiopia: High content of αs1-CN and its association with coagulation properties, whey syneresis and micelle size. Small Ruminant Res. 119, 146–155. 10.1016/j.smallrumres.2014.02.011

[B123] Mestawet T. A. Girma A. Adnoy T. Devold T. G. Vegarud G. E. (2013). Newly identified mutations at the CSN1S1 gene in Ethiopian goats affect casein content and coagulation properties of their milk. J. Dairy Sci. 96, 4857–4869. 10.3168/jds.2012-6467 23706484

[B124] Miocinovic J. Miloradovic Z. Josipovic M. Nedeljkovic A. Radovanovic M. Pudja P. (2016). Rheological and textural properties of goat and cow milk set type yoghurts. Int. Dairy J. 58, 43–45. 10.1016/j.idairyj.2015.11.006

[B125] Moatsou G. Molle D. Moschopoulou E. Valerie G. (2007). Study of caprine beta-casein using reversed-phase high-performance liquid chromatography and mass spectroscopy: Identification of a new genetic variant. Protein J. 26, 562–568. 10.1007/s10930-007-9098-8 17846874

[B126] Moatsou G. Samolada M. Panagiotou P. Anifantakis E. (2004). Casein fraction of bulk milks from different caprine breeds. Food Chem. 87, 75–81. 10.1016/j.foodchem.2003.10.020

[B127] Molik E. Bonczar G. Misztal T. Żebrowska A. Zięba D. (2012). “The effect of the photoperiod and exogenous melatonin on the protein content in sheep milk,” in Milk protein. Editor Wl H. (Croatia: InTechOpen), 352.

[B128] Neveu C. Molle D. Moreno J. Martin P. Leonil J. (2002). Heterogeneity of caprine beta-casein elucidated by RP-HPLC/MS: Genetic variants and phosphorylations. J. Protein Chem. 21, 557–567. 10.1023/a:1022433823559 12638658

[B129] Nguyen H. T. H. Afsar S. Day L. (2018). Differences in the microstructure and rheological properties of low-fat yoghurts from goat, sheep and cow milk. Food Res. Int. 108, 423–429. 10.1016/j.foodres.2018.03.040 29735076

[B130] Nicolai M. A. Garro G. Caira S. Mauriello R. Quarto M. Pascale S. D. (2021). Occurrence of quantitative genetic polymorphism at the caprine β-CN locus, as determined by a proteomic approach. Int. Dairy J. 112, 104855. 10.1016/j.idairyj.2020.104855

[B131] O'mahony J. A. Fox P. F. Boland H. S. Othman O. E. Ahmed S. (2014). Milk: An OverviewAnalysis of genetic polymorphisms in the Egyptian goats CSN1S2 using polymerase chain reaction. J. Biol. Sci. 6, 238–241.

[B132] Ozdemir M. Kopuzlu S. Topal M. Bilgin O. C. (2018). Relationships between milk protein polymorphisms and production traits in cattle: A systematic review and meta-analysis. Arch. Anim. Breed. 61, 197–206. 10.5194/aab-61-197-2018

[B133] Palmeri M. Mastrangelo S. Sardina M. T. Portolano B. (2014). Genetic variability at *α*s_2_ *-casein* gene in *Girgentana* dairy goat breed. Italian J. Animal Sci. 13, 2997. 10.4081/ijas.2014.2997

[B134] Park Y. W. (2009). Bioactive components in milk and dairy products. Hoboken, NJ, USA: Wiley.

[B135] Park Y. W. Haenlein G. F. W. (2017). “Therapeutic, hypo-allergenic and bioactive potentials of goat milk, and manifestations of food allergy,” in Handbook of milk of non-bovine mammals, 151–179.

[B136] Park Y. W. Haenlein G. F. W. Wendorff W. L. (2017). Handbook of milk of non-bovine mammals. Hoboken, NJ, USA: Wiley.

[B137] Park Y. W. Juárez M. Ramos M. Haenlein G. F. W. (2007). Physico-chemical characteristics of goat and sheep milk. Small Ruminant Res. 68, 88–113. 10.1016/j.smallrumres.2006.09.013

[B138] Persuy M. A. Printz C. Medrano J. F. Mercier J. C. (1999). A single nucleotide deletion resulting in a premature stop codon is associated with marked reduction of transcripts from a goat beta-casein null allele. Anim. Genet. 30, 444–451. 10.1046/j.1365-2052.1999.00547.x 10612234

[B139] Pirisi A. Colin O. Laurent F. Scher J. Parmentier M. (1994). Comparison of milk composition, cheesemaking properties and textural characteristics of the cheese from two groups of goats with a high or low rate of αS1-casein synthesis. Int. Dairy J. 4, 329–345. 10.1016/0958-6946(94)90030-2

[B140] Prinzenberg E. M. Gutscher K. Chessa S. Caroli A. Erhardt G. (2005). Caprine kappa-casein (CSN3) polymorphism: New developments in molecular knowledge. J. Dairy Sci. 88, 1490–1498. 10.3168/jds.S0022-0302(05)72817-4 15778318

[B141] Quaglia G. B. Gennaro L. (2003). “Enzymes uses in food processing,” in Encyclopedia of food sciences and nutrition. Editor Caballero B.. Second edition (Oxford: Academic Press), 2125–2139.

[B142] Rahmatalla S. A. Arends D. Said Ahmed A. Hassan L. M. A. Krebs S. Reissmann M. (2021). Capture sequencing to explore and map rare casein variants in goats. Front. Genet. 12, 620253. 10.3389/fgene.2021.620253 33708238 PMC7940697

[B143] Ramunno L. Cosenza G. Gallo D. Illario R. Rando A. Masina P. (2002). Un nuovo allele al locus CSN1S1 di capra, CSN1S1 NXV Congr, 221.

[B144] Ramunno L. Cosenza G. Pappalardo M. Longobardi E. Gallo D. Pastore N. (2001a). Characterization of two new alleles at the goat CSN1S2 locus. Anim. Genet. 32, 264–268. 10.1046/j.1365-2052.2001.00786.x 11683712

[B145] Ramunno L. Cosenza G. Rando A. Illario R. Gallo D. Berardino D. D. (2004). The goat alphas1-casein gene: Gene structure and promoter analysis. Gene 334, 105–111. 10.1016/j.gene.2004.03.006 15256260

[B146] Ramunno L. Cosenza G. Rando A. Pauciullo A. Illario R. Gallo D. (2005). Comparative analysis of gene sequence of goat CSN1S1 F and N alleles and characterization of CSN1S1 transcript variants in mammary gland. Gene 345, 289–299. 10.1016/j.gene.2004.12.003 15716101

[B147] Ramunno L. Longobardi E. Pappalardo M. Rando A. Di Gregorio P. Cosenza G. (2001b). An allele associated with a non-detectable amount of alpha s2 casein in goat milk. Anim. Genet. 32, 19–26. 10.1046/j.1365-2052.2001.00710.x 11419340

[B148] Ramunno L. Rando A. Di Gregorio P. Massari M. Blasi M. Masina P. (1991). “Struttura genetica di alcune popolazioni caprine allevate in Italia a locus della caseina αs1,” in IX Congress Nazionales ASPA, Milan, Italy.

[B184] Rando A. (1998). GenBank Accession no. AJ011018 [Capra hircus *CSN2* gene, exons 1 to 9, allele A]. Available at: http://www.ncbi.nlm.nih.gov/nuccore/AJ011018 (Accessed October 21, 2020).

[B149] Rando A. Pappalardo M. Capuano M. Di Gregorio P. Ramunno L. (1996). Two mutations might be responsible for the absence of b-casein in goat milk. Anim. Genet. 27, 31.

[B150] Rando A. Ramunno L. Masina P. (2000). Mutations in casein genes. Zoot. Nutr. Anim. 26, 105–114.

[B151] Roberts B. Ditullio P. Vitale J. Hehir K. Gordon K. (1992). Cloning of the goat beta-casein-encoding gene and expression in transgenic mice. Gene 121, 255–262. 10.1016/0378-1119(92)90129-d 1446822

[B152] Robinson F. (2011). Goats milk - a suitable hypoallergenic alternative? Br. Food J. 103, 198–208. 10.1108/00070700110386746

[B153] Robinson F. (2001). Goats milk – A suitable hypoallergenic alternative? Br. Food J. 103, 198–208. 10.1108/00070700110386746

[B154] Rout P. K. Kumar A. Mandal A. Laloe D. Singh S. K. Roy R. (2010). Characterization of casein gene complex and genetic diversity analysis in Indian goats. Anim. Biotechnol. 21, 122–134. 10.1080/10495390903534622 20379889

[B155] Sacchi P. Chessa S. Budelli E. Bolla P. Ceriotti G. Soglia D. (2005). Casein haplotype structure in five Italian goat breeds. J. Dairy Sci. 88, 1561–1568. 10.3168/jds.S0022-0302(05)72825-3 15778326

[B156] Sadler M. J. Smith N. (2013). Beta-casein proteins and infant growth and development. infant 9, 173–176.

[B157] Schmidely P. Meschy F. Tessier J. Sauvant D. (2002). Lactation response and nitrogen, calcium, and phosphorus utilization of dairy goats differing by the genotype for alpha S1-casein in milk, and fed diets varying in crude protein concentration. J. Dairy Sci. 85, 2299–2307. 10.3168/jds.S0022-0302(02)74310-5 12362463

[B158] Selvaggi M. Laudadio V. Dario C. Tufarelli V. (2014). Major proteins in goat milk: An updated overview on genetic variability. Mol. Biol. Rep. 41, 1035–1048. 10.1007/s11033-013-2949-9 24381104

[B159] Selvaggi M. Tufarelli V. (2011). “Chapter 1. Caseins of goat and sheep milk: Analytical and technological aspects,” in Casein: Production, uses and health effects. Editors Ventimiglia A. M. Birkenhager J. M. (New York: Nova Science Publishers), 1–25.

[B160] Shekar P. C. Goel S. Rani S. D. Sarathi D. P. Alex J. L. Singh S. (2006). kappa-casein-deficient mice fail to lactate. Proc. Natl. Acad. Sci. U. S. A. 103, 8000–8005. 10.1073/pnas.0601611103 16698927 PMC1472419

[B161] Silva S. V. Pihlanto A. Malcata F. X. (2006). Bioactive peptides in ovine and caprine cheeselike systems prepared with proteases from Cynara cardunculus. J. Dairy Sci. 89, 3336–3344. 10.3168/jds.S0022-0302(06)72370-0 16899666

[B162] Simões J. Gutiérrez C. (2018). Sustainable goat production in adverse environments: Volume I: Welfare, health and breeding. Berlin, Germany: Springer International Publishing.

[B163] Strzelec E. Niżnikowski R. (2011). SNPs polymorphisms in LGB, CSN3 and GHR genes in five goat breeds kept in Poland. Animal Sci. 49, 181–188.

[B164] Swaisgood H. E. (2003). “Chemistry of the caseins,” in Advanced dairy chemistry—1 proteins: Part A/Part B. Editors Fox P. F. Mcsweeney. P. L. H. (Boston: Springer US), 139–201.

[B165] Tadlaoui Ouafi A. Babilliot J. M. Leroux C. Martin P. (2002). Genetic diversity of the two main Moroccan goat breeds: Phylogenetic relationships with four breeds reared in France. Small Ruminant Res. 45, 225–233. 10.1016/S0921-4488(02)00111-6

[B166] Tailford K. A. Berry C. L. Thomas A. C. Campbell J. H. (2003). A casein variant in cow's milk is atherogenic. Atherosclerosis 170, 13–19. 10.1016/S0021-9150(03)00131-X 12957678

[B167] Threadgill D. W. Womack J. E. (1990). Genomic analysis of the major bovine milk protein genes. Nucleic Acids Res. 18, 6935–6942. 10.1093/nar/18.23.6935 1979856 PMC332753

[B168] Tortorici L. Di Gerlando R. Mastrangelo S. Sardina M. T. Portolano B. (2014). Genetic characterisation of CSN2 gene in Girgentana goat breed. Italian J. Animal Sci. 13, 3414. 10.4081/ijas.2014.3414

[B169] Vacca G. M. Ali H. O. Carcangiu V. Pazzola M. Dettori M. L. (2009a). Genetic structure of the casein gene cluster in the Tunisian native goat breed. Small Ruminant Res. 87, 33–38. 10.1016/j.smallrumres.2009.09.034

[B170] Vacca G. M. Ali H. O. Pazzola1 M. Dettori M. L. Carcangiu V. (2009b). An investigation on allele frequency at the CSN1S2 locus and its relationship with milk parameters in the Sarda goat. J. Anim. Feed Sci. 18, 628–637. 10.22358/jafs/66437/2009

[B171] Vacca G. M. Dettori M. L. Piras G. Manca F. Paschino P. Pazzola M. (2014). Goat casein genotypes are associated with milk production traits in the Sarda breed. Anim. Genet. 45, 723–731. 10.1111/age.12188 24990661

[B172] Vacca-Smith A. M. Bowen W. H. (1995). The effect of milk and kappa casein on streptococcal glucosyltransferase. Caries Res. 29, 498–506. 10.1159/000262121 8556755

[B173] Veltri C. Lagonigro R. Pietrolà E. D’andrea M. Pilla F. Chianese L. (2000). “Molecular characterization of the goat αs2-casein E allele and its detection in goat breeds of Italy,” in Proceedings 7th International Conference on Goats, Tour, France.

[B174] Wal J.-M. (1998). Cow's milk allergens. Allergy 53, 1013–1022. 10.1111/j.1398-9995.1998.tb03811.x 9860234

[B175] Wal J. M. (2002). Cow's milk proteins/allergens. Ann. Allergy Asthma Immunol. 89, 3–10. 10.1016/s1081-1206(10)62115-1 12487197

[B176] Wang Q. Huang Z. Chen M. J. Huang S. Z. Zeng Y. T. (2001). GenBank Accession no. AF409096 [*Capra hircus* β-casein precursor (csn2) gene, complete cds]. Available at: http://www.ncbi.nml.nih.gov (Accessed Feb 01, 2021).

[B177] Yahyaoui M. H. Angiolillo A. Pilla F. Sanchez A. Folch J. M. (2003). Characterization and genotyping of the caprine kappa-casein variants. J. Dairy Sci. 86, 2715–2720. 10.3168/jds.S0022-0302(03)73867-3 12939096

[B178] Yahyaoui M. H. Coll A. Sanchez A. Folch J. M. (2001). Genetic polymorphism of the caprine kappa casein gene. J. Dairy Res. 68, 209–216. 10.1017/s0022029901004733 11504385

[B179] Yangilar F. (2013). As a potentially functional food: Goats¡¯ milk and products. J. Food Nutr. Res. 1, 68–81. 10.12691/jfnr-1-4-6

[B180] Yue X. P. Fang Q. Zhang X. Mao C. C. Lan X. Y. Chen H. (2013). Effects of CSN1S2 genotypes on economic traits in Chinese dairy goats. Asian-Australas. J. Anim. Sci. 26, 911–915. 10.5713/ajas.2013.13018 25049867 PMC4093498

[B181] Zhang Y. Chen R. Ma H. Chen S. (2015). Isolation and identification of dipeptidyl peptidase IV-inhibitory peptides from trypsin/chymotrypsin-treated goat milk casein hydrolysates by 2D-TLC and LC–MS/MS. J. Agric. Food Chem. 63, 8819–8828. 10.1021/acs.jafc.5b03062 26323964

[B182] Zucht H.-D. Raida M. Adermann K. Mägert H.-J. Forssmann W.-G. (1995). Casocidin-I: A casein-αs2 derived peptide exhibits antibacterial activity. FEBS Lett. 372, 185–188. 10.1016/0014-5793(95)00974-e 7556666

[B183] Zullo A. Barone C. M. A. Chianese L. Colatruglio P. Occidente M. Matassino D. (2005). Protein polymorphisms and coagulation properties of Cilentana goat milk. Small Ruminant Res. 58, 223–230. 10.1016/j.smallrumres.2004.10.003

